# Opposite response modes of NADW dynamics to obliquity forcing during the late Paleogene

**DOI:** 10.1038/s41598-020-70020-2

**Published:** 2020-08-06

**Authors:** Hojun Lee, Kyoung-nam Jo, Sangmin Hyun

**Affiliations:** 1 Division of Geology and Geophysics, College of Natural Sciences, Kangwon National University, 1 Kangwondaehak-gil, Chuncheon-si, Gangwon-do 24341 Republic of Korea; 2 Marine Environmental Research Center, Korea Institute of Ocean Science and Technology, Haeyang-ro 385, Yeongdo-gu, Busan 49111 Republic of Korea

**Keywords:** Palaeoceanography, Palaeoclimate

## Abstract

Although the responses of North Atlantic Deep Water (NADW) is deeply connected to orbital rhythms, those under different tectonic and atmospheric boundary conditions remain unknown. Here, we report suborbitally resolved benthic foraminiferal stable isotope data from J-anomaly Ridge in the North Atlantic from ca. 26.4–26.0 Ma. Our results indicate that the formation of NADW during that time interval was increased during the obliquity-paced interglacial periods, similar to in the Plio-Pleistocene. During the late Oligocene, the interglacial poleward shifts of the stronger westerlies in the southern hemisphere, which occurred due to the higher thermal contrasts near the upper limit of the troposphere, reinforced the Antarctic Circumpolar Current (ACC) and, in turn, the Atlantic meridional overturning circulation (AMOC). However, such a response mode in deep ocean circulation did not occur during the middle Eocene because of different tectonic boundary conditions and the immature states of the ACC. Instead, the middle Eocene interglacial conditions weakened the formation of the proto-type NADW due to less heat loss rate in high-latitude regions of the North Atlantic during high obliquity periods. Our findings highlight the different responses of deep ocean circulation to orbital forcing and show that climate feedbacks can be largely sensitive to boundary conditions.

## Introduction

Atlantic meridional overturning circulation (AMOC) has played a crucial role in internal climate feedbacks throughout the past several tens of million years, in terms of the redistribution of heat and greenhouse gasses e.g.^1^. Earth’s orbital configurations (precession, obliquity, and eccentricity) are regarded as key modulators of the strength of the AMOC. For example, the formation of North Atlantic Deep Water (NADW) was more vigorous during interglacial periods than glacial periods, corresponding to the obliquity periodicity in the recent geologic past, such as during the Plio-Pleistocene^2,3^. Although paleoceanographic changes paced with obliquity cycles have been proven even for earlier geologic times, such as the Paleogene^4–6^, the fundamental mechanism linking orbital forcing with deep ocean circulations remains unclear.

The gradual strengthening of the AMOC resulted from the expansion of the Antarctic Circumpolar Current (ACC) throughout the middle to late Paleogene^7^. Previous studies have also suggested strong influences of the ACC on both the thermal isolation of the high-latitude Southern Ocean and NADW formation^8,9^. Modeling results suggest that the ACC is one of the major drivers of current NADW dynamics^10,11^. Thus, high-resolution paleoceanographic records from a key area of AMOC during the middle to late Paleogene (when the ACC evolved) could provide new insight into NADW dynamics.

**Figure 1:**
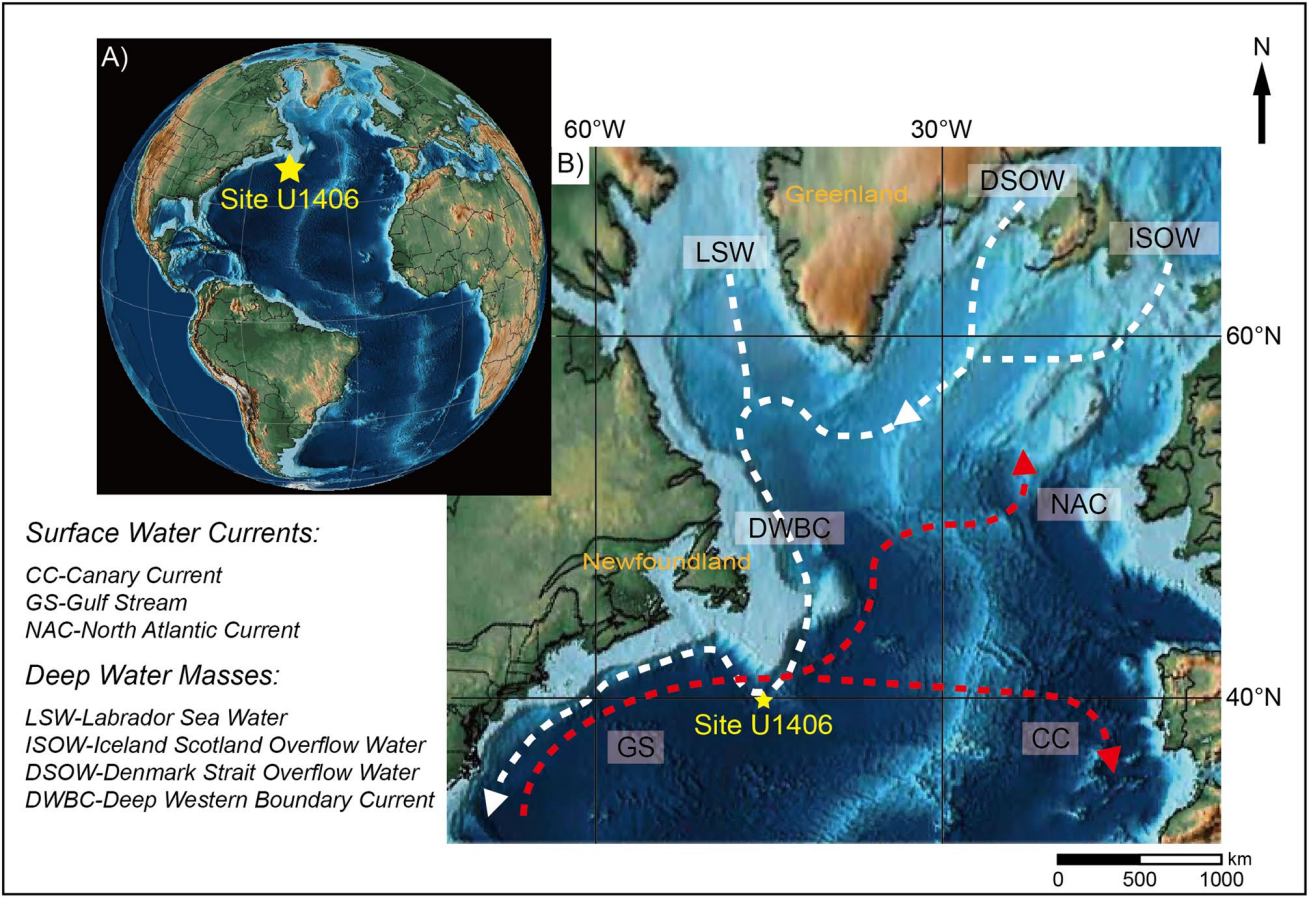
Location and oceanographic setting of Integrated Ocean Drilling Program (IODP) Expedition 342 Site U1406 in the late Oligocene plate reconstruction map created using the open source software Gplates 2.1 (https://gplates.org) with the tectonic model from Ref. ^47^. (**A**) The core site is located in the western North Atlantic. (**B**) The study area lies under the pathway of the deep western boundary current (DWBC) originating from the high-latitude North Atlantic Ocean. Flow directions of surface and deep water currents are indicated by the red and white arrows, respectively.

Integrated Ocean Drilling Program (IODP) Expedition 342 Site U1406 (40° 20.99′ N, 51° 38.99′ W; 3,814 m below current mean sea level), located in J-anomaly Ridge in the North Atlantic, is considered as a suitable area for estimating the NADW dynamics because of its geographical position (Fig. 1): directly affected by the deep western boundary current (DWBC), a geostrophic current of the NADW, while the sea surface area of this core site is affected by the Gulf Stream in the North Atlantic subtropical gyre^12,13^. In addition, the DWBC facilitates the orbital-scale paleoceanographic study of the Oligocene based on analysis of sediment drift, which was deposited at a higher sedimentation rate (≥ 2 cm/kyr) compared to other pelagic sites. The foraminiferal tests are relatively well preserved in this sediment, due to its impermeable nature inhibiting interaction between shells and pore water^14,15^. Also, the relatively shallow burial depth of the Paleogene deposit, covered by a thin Neogene sedimentary layer, caused by the NADW strengthening that occurred after the Oligocene could minimize early diagenetic effects on the calcium carbonate (CaCO_3_) sediments^12,14^. These geological features of the drilling site allow us to shed new light on orbital-scale variations of NADW during the Paleogene in the mid-latitude North Atlantic Ocean.

In this study, we report suborbital-scale benthic foraminiferal isotope records with additional proxy data during the late Oligocene from IODP Site U1406. Ultimately, these records are compared to previous results from the same area during the middle Eocene, to delineate orbital-scale NADW dynamics before and after the ACC.

## Results

**Figure 2:**
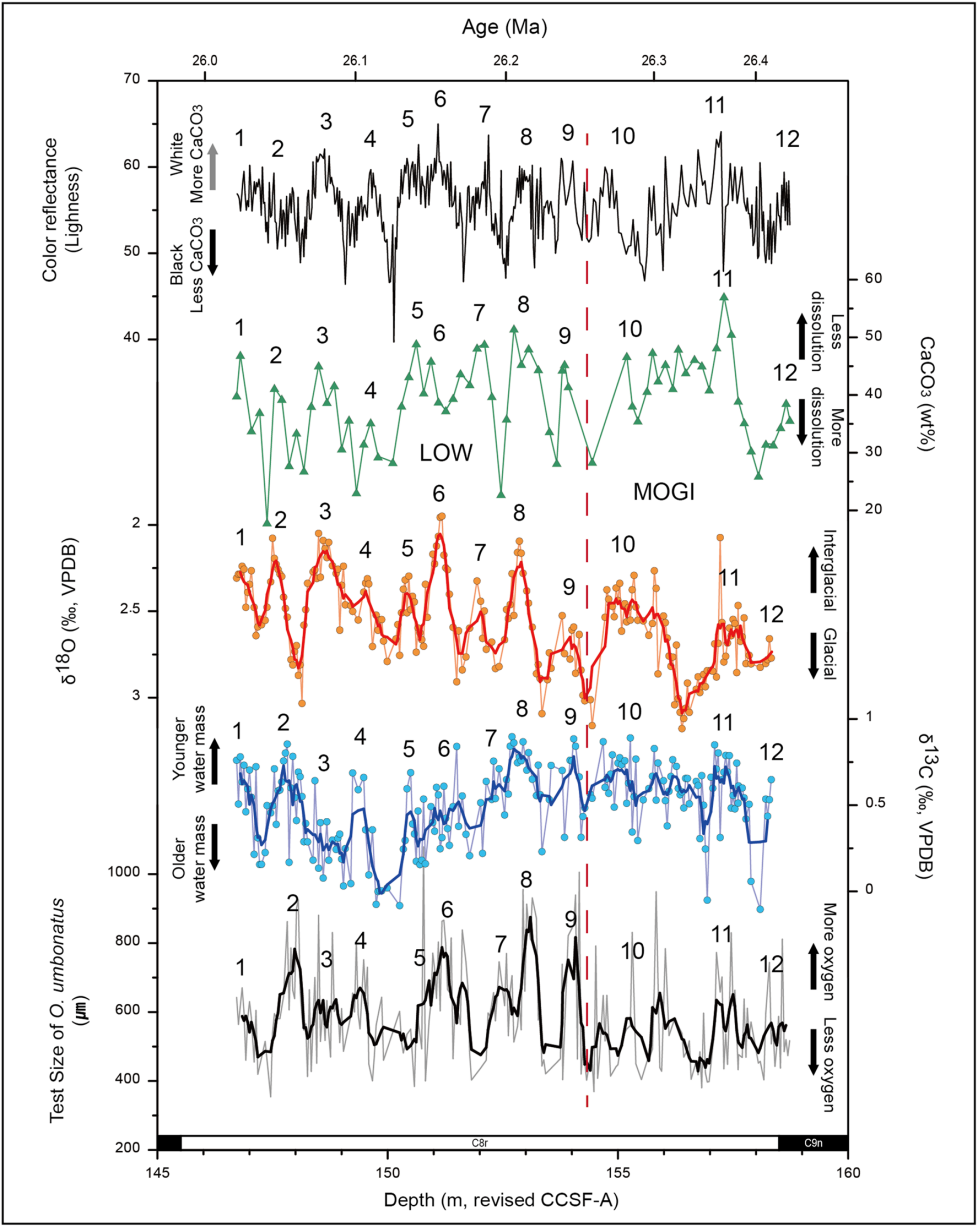
Benthic foraminiferal δ^18^O, δ^13^C, *O. umbonatus* test sizes, calcium carbonate (CaCO_3_) contents, and color reflectance versus depth. The paleomagnetic polarity reversals (bottom) and age (top) are also shown. The five-point moving-averaged data of δ^18^O, δ^13^C, and test size are represented by the bold lines in each profile. The vertical dashed red line indicates the boundary between the Late Oligocene Warming (LOW) and Mid-Oligocene Glacial Interval (MOGI), as suggested by Ref.^16^. Each record shows very similar short-term variability (labeled by number), related to the obliquity-paced glacial–interglacial cycles based on the δ^18^O record (see the main text and Supplemental Materials).

Based on the preexisting age models, the ages of study interval (146.72–158.73 m) were calculated to be 26.02–26.43 Ma (Supplementary Fig. S1; see methods). Our benthic foraminiferal δ^18^O record revealed a long-term gradual decrease of 0.4‰ between ca. 26.25 Ma (154.3 m) and 26.02 Ma (Fig. 2). The δ^13^C values also gradually decreased by 0.5‰ between 26.25 and 26.12 Ma (150.0 m), and then recovered. The onset of long-term changes in both isotope records coincides with the boundary between the Mid Oligocene Glacial Interval (MOGI; 26.3 to 28.0 Ma by Ref.^16^) and the Late Oligocene Warming (LOW; 23.7 to 26.3 Ma by Ref.^16^). The long-term patterns of both δ^18^O and δ^13^C are in accordance with those of other Atlantic and Pacific sites during the same period, and they are considered to be related to ~ 405-kyr eccentricity cycles^4,5,17^. Short-term fluctuations of 0.5–1.0‰, with prominent quasi-periodicities, are superimposed on the long-term trends of the δ^18^O and δ^13^C records.

These isotopic periodicities correlate very well with additional proxy records, such as foraminiferal test size and CaCO_3_ contents data, with only a few discordant peaks (Fig. 2). In this study, the onboard color reflectance data (L*), which are considered useful for estimating relative changes in CaCO_3_ contents^12,18^, were used to verify the periodicity based on the relatively low-resolution CaCO_3_ content data. The results of spectral analyses of the δ^18^O, δ^13^C, L*, and *O. umbonatus* test size data indicate similar periodicities of 0.95, 1.15, 0.95, and 1.0 m, respectively (Supplementary Fig. S2).

## Discussion

The concordance of the periodicities among all of proxy records may indicate that a single paleoceanographic factor ultimately caused the short-term fluctuation in each proxy record from 26.02–26.43 Ma. Considering an average sedimentation rate of 2.95 cm/kyr, the periodicity of our proxy records can be considered as approximately 33 kyr (0.95 m) to 39 kyr (1.15 m), which corresponds to obliquity-related periodicities (29–55 kyrs; Ref.^6,19^) (Supplementary Fig. S2). These periodicities were previously reported in the same area (IODP Site U1410) for the middle Eocene (43.5–46.0 Ma)^6^. Based on the results of spectral analysis of the L* data for the entire LOW period (24.0–26.5 Ma)^12^, the periodicity of 0.92 m still fell within the obliquity cycle of 45 kyrs (Supplementary Fig. S3), even when the age model is more robust for the longer time period^20^. Thus, our suborbital-scale records support previous results showing that periodical changes in paleoceanographic conditions in the NADW pathway were mainly controlled by the obliquity cycles^6^. Obliquity-paced paleoceanographic changes during the late Oligocene were also identified in other North Atlantic and Pacific sites, and interpreted as glacial–interglacial cycles^4,5,21^. We estimated an average temperature change of ~ 1.7 °C between the glacial and interglacial cycles, based on comparison with a previous result from the high-latitude Southern Ocean site showing minor temperature variations (see supplementary materials).

Our proxy data clearly illustrate that the late Oligocene NADW was strengthened during the interglacial periods, as shown by the lower δ^18^O values. This is because the lower δ^18^O values (an average value of ~ 2.3‰) coincided with higher δ^13^C values (an average value of ~ 0.7‰), *O. umbonatus* test sizes (an average value of ~ 708.2 μm), and CaCO_3_ contents (an average value of ~ 46.1 wt%), which can be interpreted as synchronous increases in the supply of younger water, the oxygen level of deep water, and the concentration of CO_3_^2−^ ions, respectively (Fig. 2). The higher δ^13^C values indicate younger deep water due to its shorter reaction time with organic matter^22^, where this process could reduce carbonate dissolution in the area. In addition, the stronger NADW likely supplied more oxygenated water to site U1406. Because oxygen in the deep sea is supplied by deep water circulation^23,24^, we can refer to the test sizes of the benthic foraminifera *O. umbonatus* to trace past changes in deep water circulation, where *O. umbonatus* is known to precipitate larger tests in an oxygen-enriched environment^25^. These sequential interpretations of our proxy data largely accord with those for NADW dynamics in the Plio-Pleistocene e.g.^2,3^.

**Figure 3:**
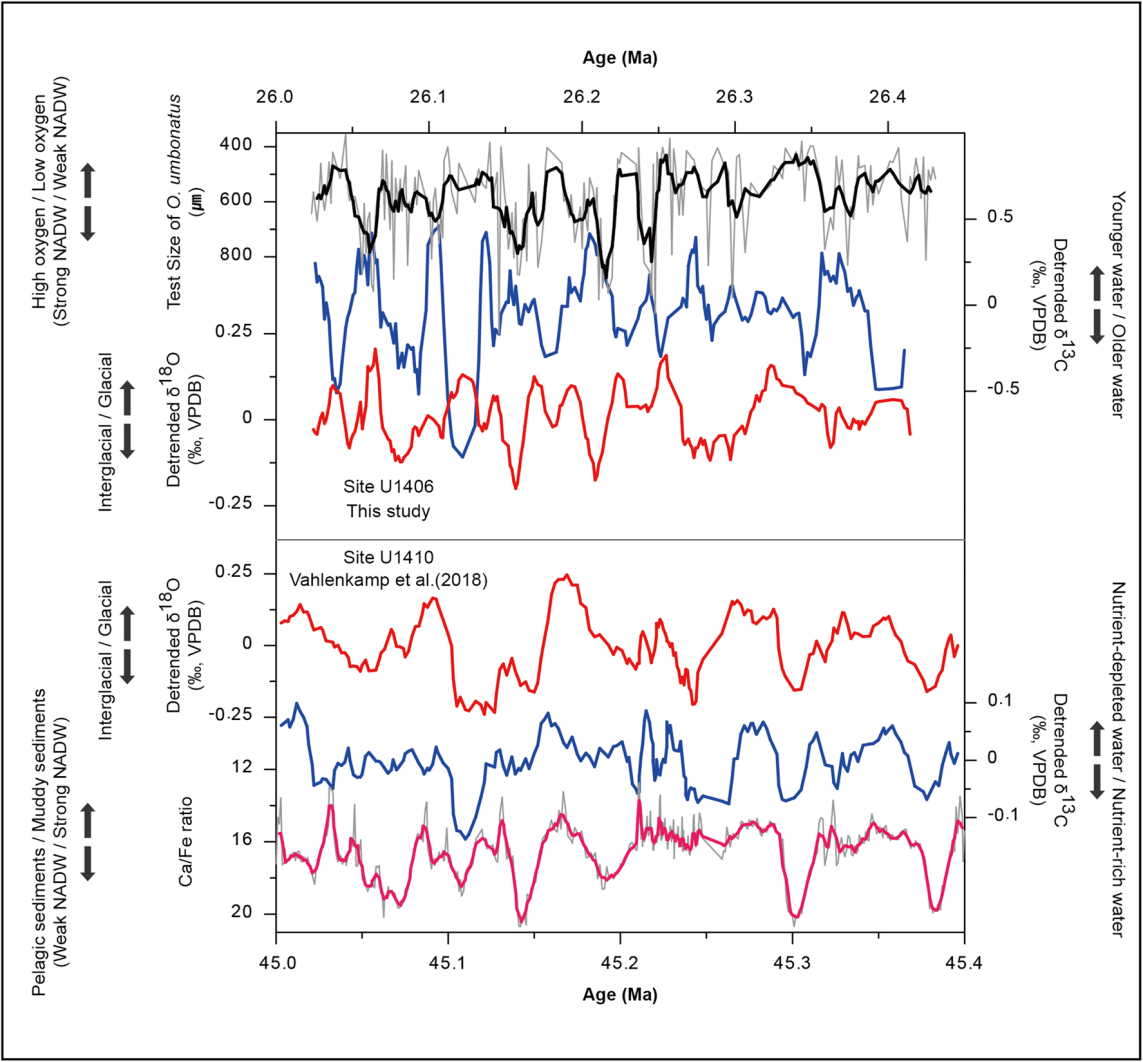
Comparison of the proxy records from the same study area for a ~ 400-kyr period between the late Oligocene (this study) and the middle Eocene^6^. Note that the δ^18^O (red) and δ^13^C (blue) data are detrended to remove long-term trends. Test sizes of *O. umbonatus* and Ca/Fe are depicted along with five-point moving-averaged data, represented by black and pink lines, respectively. The δ^18^O values and both proxy records (δ^13^C and test sizes) for the North Atlantic Deep Water (NADW) production rate for the late Oligocene show the anti-phase relationship, while those in the middle Eocene show the in-phase relationship.

Most interestingly, the relationship between the late Oligocene glacial-interglacial cycles and the relative production rate of NADW was opposite to that seen at the study site during the middle Eocene (Fig. 3). Previous researchers proposed that, based on middle Eocene Ca/Fe data from site U1410, the antecedent of the NADW (the Northern Component Water; NCW) circulation was stronger during glacial periods coinciding with obliquity periodicities^6^. They observed that the δ^13^C record shows an in-phase pattern with the δ^18^O record during the period (Fig. 3), and the changes in δ^13^C values were interpreted as arising from the variability in the supply rate of the nutrient-depleted water mass originating from the tropical surface area. As a result, during middle Eocene glacial periods with higher δ^18^O values, the higher δ^13^C values in the deep water are compatible with the vigorous NADW, which is supported by the fact that lower Ca/Fe values indicate higher rates of NADW production.

**Figure 4:**
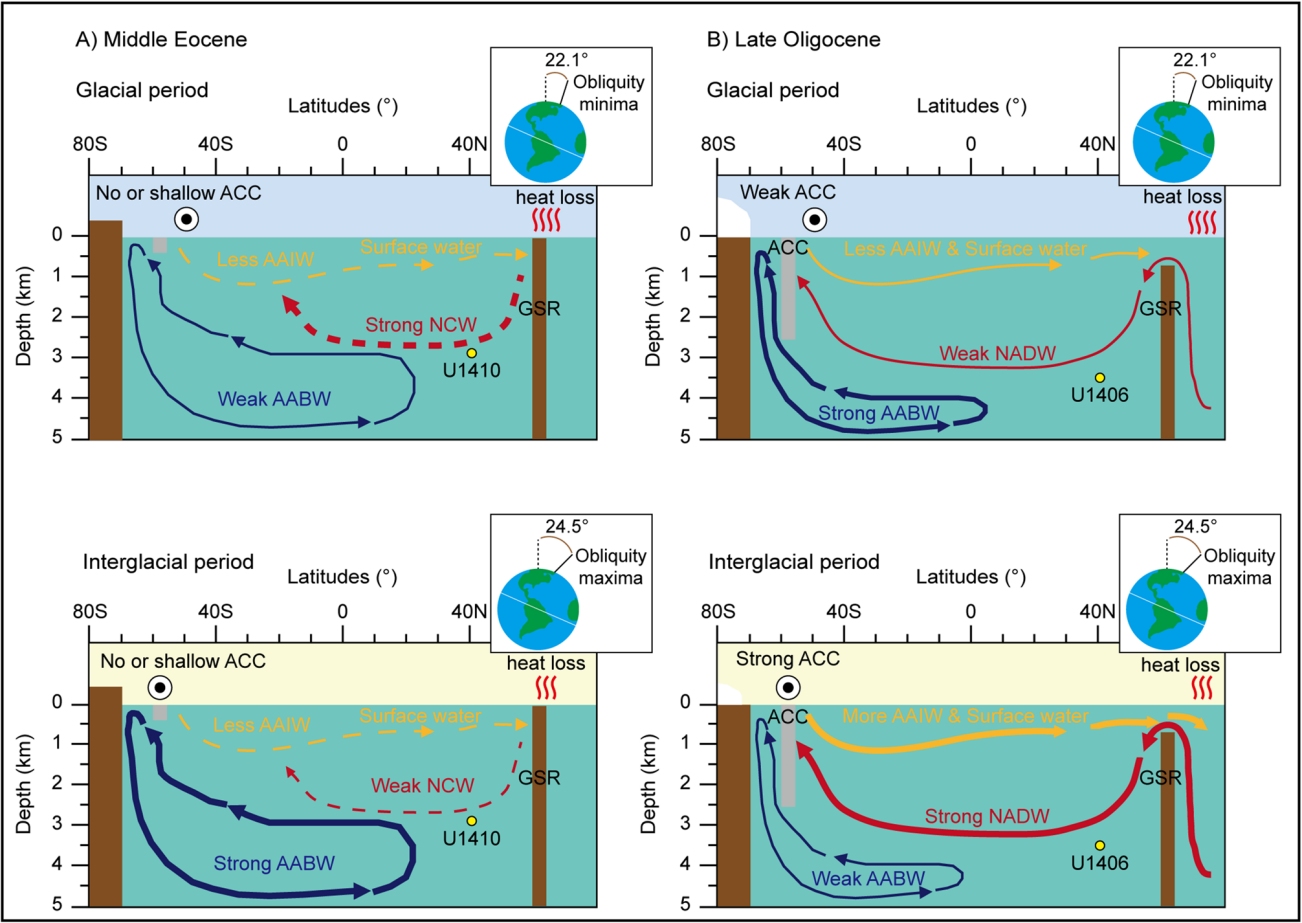
Schematic representations of the different modes of AMOC during the middle Eocene and late Oligocene. The basal structure of AMOC is based on Ref.^7^. (**A**) The obliquity-paced glacial (upper) and interglacial (lower) modes of AMOC during the middle Eocene without the ACC, Antarctic ice sheets, or GSR subduction. Note that the formation of NCW was more vigorous during the glacial period^6^. (**B**) The obliquity-paced glacial (upper) and interglacial (lower) modes of AMOC during the late Oligocene with the ACC, Antarctic ice sheets, and GSR subduction. Note that the NADW was more vigorous due to the poleward movement of westerly wind currents during the interglacial period. At the same time, the formation of the Antarctic bottom water (AABW) was relatively decreased by shrinkage of the Antarctic ice sheets and sea ice^33,48^. AABW, Antarctic Bottom Water; AAIW, Antarctic Intermediate Water; ACC, Antarctic Circumpolar Current; NADW, North Atlantic Deep Water; NCW, Northern Component Water; GSR, Greenland-Scotland Ridge.

Why do NADW dynamics respond oppositely to the same obliquity-paced glacial–interglacial cycles in the study area during different time periods in the late Paleogene? This phenomenon can be explained by the maturation of the AMOC with the Greenland-Scotland Ridge (GSR) subduction^26^, and the opening of ocean gateways e.g.^27,28^ between the middle Eocene and the late Oligocene (Supplementary Fig. S4). These major tectonic events eventually permitted completion of the ACC and strengthening of the NADW^7,29,30^ (Fig. 4). During the late Oligocene, the mature AMOC was more likely to amplify the effect of deep water reaction time on δ^13^C variability compared to the supply rate of the nutrient-depleted water although both the controlling factors on δ^13^C values could show same results eventually.

Based on our supporting evidence, the obliquity-paced glacial–interglacial cycles during the late Oligocene were very likely to affect NADW dynamics e.g.^31^. The sufficiently deepened ACC during the late Oligocene promoted the AMOC^7,32^. The strength of the modern ACC is controlled by the intensity and position of the southern westerly wind^33^. Thus, we argue that, at the late Oligocene obliquity maxima (interglacial periods), the poleward movement of the westerly wind field enhanced the upwelling of deep water originating from the NADW in the high-latitude Southern Ocean, which could have strengthened the AMOC (Fig. 4). The vigorous upwelling of CO_2_-rich deep water might have created a positive feedback loop between greenhouse gases and deep ocean circulation, because the increases in atmospheric CO_2_ concentrations could move the westerly wind field further to the south^34^. In addition, the proxy data from IODP Site U1356, off-shore of the Wilkes Land margin, shows the more vigorous upwelling of the North Atlantic sourced deep water for the obliquity-paced interglacial periods during the late Oligocene (~ 26.0–25.0 Ma)^35^. Warmer and saltier surface currents for the subduction of NADW in the high-latitude North Atlantic during interglacial periods could be introduced by the weakening of the zonal flow in the northern polar gyre, and by expansion of the subtropical gyre^36,37^.

On the other hand, it is plausible that the middle Eocene AMOC behaved differently during the commeasurable obliquity-paced glacial–interglacial cycles. Insufficient extension of ocean gateways during that time period limited the ACC flow to shallower depths than the threshold depth for regulating the AMOC e.g.^38^, although there are conflicts among the many simulation results regarding the exact threshold depth of the ACC^10,32^. A recent result suggested that the ACC occurred after 30.0 Ma^29^. Under these tectonic and oceanic conditions, the southward shift of southern westerly winds could not promote NADW formation during the middle Eocene obliquity maxima (interglacial periods) (Fig. 4). Instead, the most plausible explanation is that the Arctic cooling and partial glaciation e.g.^39^ facilitated more active formation of NADW during the middle Eocene obliquity minima (glacial periods), as suggested by Ref.^6^.

In summary, our records indicate a more vigorous NADW during the late Oligocene obliquity maxima (interglacial periods) relative to the middle Eocene. Our findings emphasize that large-scale deep ocean circulation may respond oppositely depending on the tectonic boundary conditions, in spite of equal orbital forcing.

## Methods

### Age model

The ages in this study were adjusted to the Geological Time Scale 2012 (GTS 2012^40^) by linear interpolation between paleomagnetostratigraphic and biostratigraphic records^20,41^, based on the recently revised composite depth scale of Ref.^42^. The boundaries between paleomagnetic polarity reversals are C8r/C9n (158.45 m; 26.420 Ma) and C8n.2n/C8r (145.52 m; 25.987 Ma) (Supplementary Table S1). In addition, the last appearance datum (LAD) of *Areoligera semicirculata* and *Saturnodinium pansum* dinocysts indicates 26.3 Ma (155.89 m), while that of *Enneadocysta pectiniformis* dinocysts indicates 26.7 Ma (165.59 m) (Supplementary Table S1). Based on the sedimentation rates, the age range in this study is the ~ 400-kyr period from 26.02–26.43 Ma (146.72–158.73 m) (Supplementary Fig. S1). This is almost coincident with a paleomagnetic age range of C8r, and covers the transition period from the MOGI to the earliest LOW.

### Stable isotope analysis and interspecies verifications

In this study, we reconstructed suborbital-scale (~ 2 kyr) stable isotope records (δ^18^O and δ^13^C) of benthic foraminifera. For stable isotope analysis, handpicked tests (> 250 µm) of shallow infaunal (*Oridorsalis umbonatus*) and epifaunal (*Cibicidoides* spp.) benthic foraminiferal assemblages were crushed and rinsed with methanol. After washing, dehydrated samples were analyzed using a gas-ratio mass spectrometer (Finnigan MAT 252) coupled to an automated carbonate device (KIEL-III) at the Environmental Isotope Laboratory of the University of Arizona. The results are expressed relative to Vienna Peedee Belemnite (VPDB), and the one-sigma precision is ± 0.10‰ for δ^18^O and ± 0.08‰ for δ^13^C. We analyzed both of *O. umbonatus* and *Cibicidoides* spp. in samples including both species to verify interspecies offsets. Based on the results, we added 0.14‰ and 1.19‰ to the original *O. umbonatus* δ^18^O and δ^13^C values, respectively, for compatibility with the 0.64‰-adjusted *Cibicidoides* spp. values (converted due to the vital effect)^43^ (Supplementary Fig. S5, Tables S2 and S3). These interspecies offsets are exactly coincident with the results of Ref.^43,44^.

### Calcium carbonate contents and foraminiferal test sizes

For estimating CaCO_3_ contents of 71 samples (Supplementary Table S4), we used the difference in weight between the original samples and CaCO_3_-dissolved samples. At first, we weighed the bulk sediments dried at 40 °C for 48 h, and then removed CaCO_3_ components in all samples by using 10% hydrochloric acid. After the CaCO_3_ dissolution, each residue was dried at same temperature for 48 h and weighed. Finally, we determined CaCO_3_ contents by subtracting the weight of CaCO_3_-dissolved samples from that of the original samples; the contents are presented in weight percent (wt%).

In our previous research, we determined the diameter of a single species of *O. umbonatus*, existing in the almost whole study interval (218 samples in total), under the stereoscopic microscope by using NIS-Elements software^45^ (Supplementary Table S5). According to the method of Ref.^26^, the largest *O. umbonatus* specimen in each sample was used to avoid the effect of juvenile foraminifera.

### Statistical analysis

To identify orbital periodicities from our unevenly spaced proxy records, we performed spectral analyses on the detrended stable isotope, *O. umbonatus* test size and the L* data using the open-source software REDFIT^46^ (Fig. S2). This software was devised to estimate the true spectrum of a time series without the overestimated low-frequency components by interpolation in the time domain. Thus, we can use this program to test if peaks in the spectrum of a time series are significant against the red-noise (a continuous decrease of spectral amplitude with increasing frequency) background from a first-order autoregressive (AR1) process. The input parameters used for the analysis in this study were as follows: Number of Monte-Carlo simulations = 1,000, Toggle calculation of false-alarm levels based on Monte-Carlo simulation = set to T (perform Monte-Carlo test), rhopre = − 99.0, Oversampling factor for Lomb-Scargle Fourier transform = 2, Number of WOSA segments = 4, Window-type identifier used to suppress sidelobes in spectral analysis = Blackman–Harris.

## Supplementary information

Supplementary file1

## Data Availability

All data used in this study are included in the Supplementary Materials.
