# Supplementary material for: Opposite response modes of NADW dynamics to obliquity forcing during the late Paleogene
Source: Sci Rep. 2020 Aug 6;10:13194. doi: 10.1038/s41598-020-70020-2 (PMC7413374; doi:10.1038/s41598-020-70020-2)
Supplement: Supplementary file 1 — Supplementary file1 [file 41598_2020_70020_MOESM1_ESM.pdf]

Supplemental Materials

Opposite response modes of NADW dynamics to obliquity forcing during the late Paleogene

Hojun Lee[1], Kyoung-nam Jo[1,*], and Sangmin Hyun[2]

[1]*Division of Geology and Geophysics, College of Natural Sciences, Kangwon National University, 1Kangwondaehak-gil, Chuncheon-si, Gangwon-do, 24341, Republic of Korea*

[2]*Marine Environments and Conservation Research Division, Korea Institute of Ocean Science and Technology, Haeyang-ro 385, Yeongdo-gu, Busan, 49111, Republic of Korea*

Correspondence to E-mail: kjo@kangwon.ac.kr

Supplementary figures

1. Figure S1. Age model used in this study
2. Figure S2. Results of the spectral analysis of proxy records conducted in this study
3. Figure S3. Results of the spectral analysis of changes in color reflectance data (L*) during the entire Late Oligocene Warming (LOW) period
4. Figure S4. Tectonic evolution with the stable isotope records during the Paleogene.
5. Figure S5. The adjusted interspecies $\delta^{18}O$ (upper) and $\delta^{13}C$ (lower) records

Supplementary tables

1. Table S1. Datums used in the age model of this study
2. Table S2. Oxygen isotope data of *Cibicidoides* spp. and *Oridorsalis umbonatus*
3. Table S3. Carbon isotope data of *Cibicidoides* spp. and *Oridorsalis umbonatus*
4. Table S4. Calcium carbonate ($CaCO_3$) contents in this study
5. Table S5. Test size records of *Oridorsalis umbonatus*

Temperature estimation

We estimated temperature changes between the obliquity-paced glacial and interglacial periods based on the foraminiferal $\delta^{18}O$ records regulated by the temperature and $\delta^{18}O$ of peripheral seawater. While the late Oligocene obliquity-paced variations of $\delta^{18}O$ values at site U1406 are within ~0.5–1.0 ‰, the $\delta^{18}O$ records from Ocean Drilling Program site 690 in the high-latitude Southern Ocean show changes of ~0.2–0.5 ‰ during the same time interval [1]. Previous research suggested that, based on the taxonomic analytical data of calcareous nannofossils, there was only a minor contribution of increasing temperature to $\delta^{18}O$ values at site 690 prior to 26.0 Ma [2]. Accordingly, if it can be assumed that $\delta^{18}O$ values in the high-latitude Southern Ocean site were mainly controlled by changes in the global ice volume [1, 3], the remaining difference in $\delta^{18}O$ values (0.0–0.8 ‰) is attributable to changes of 0–3.4 °C in the study area. In addition, the average of this temperature range (1.7 °C) is similar to a modeling result suggesting a glacial–interglacial temperature change of 1.2 °C at site U1410 during the middle Eocene [4], coincident with obliquity variations.

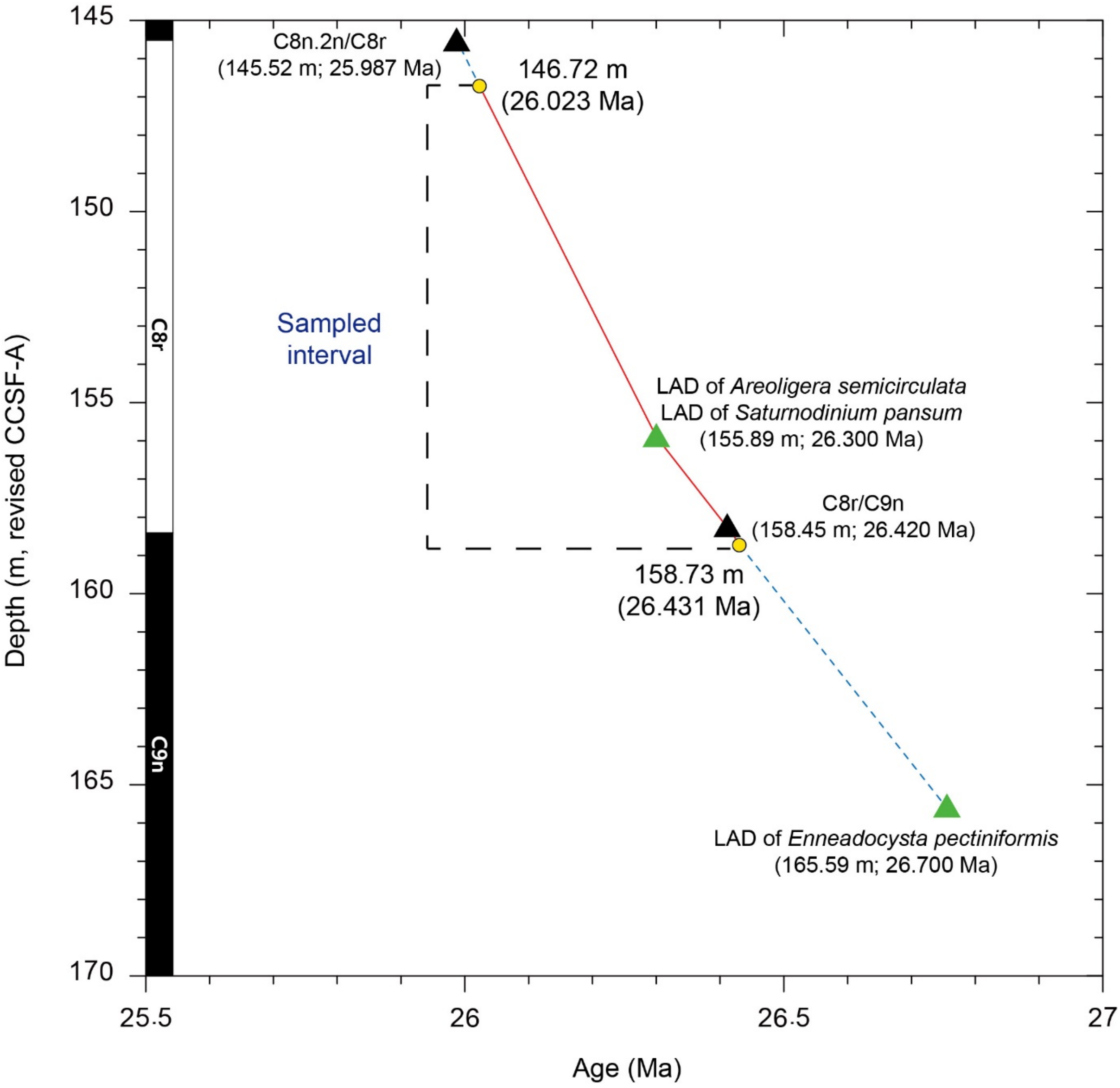

Figure S1. Age model used in this study (IODP Site U1406). This age model is based on interpolations using paleomagnetic tie points and biostratigraphic data [5, 6]. Each green triangle is the last appearance datum (LAD) of dinocysts and each black triangle is a paleomagnetic polarity reversal. The study period is indicated by the red line.

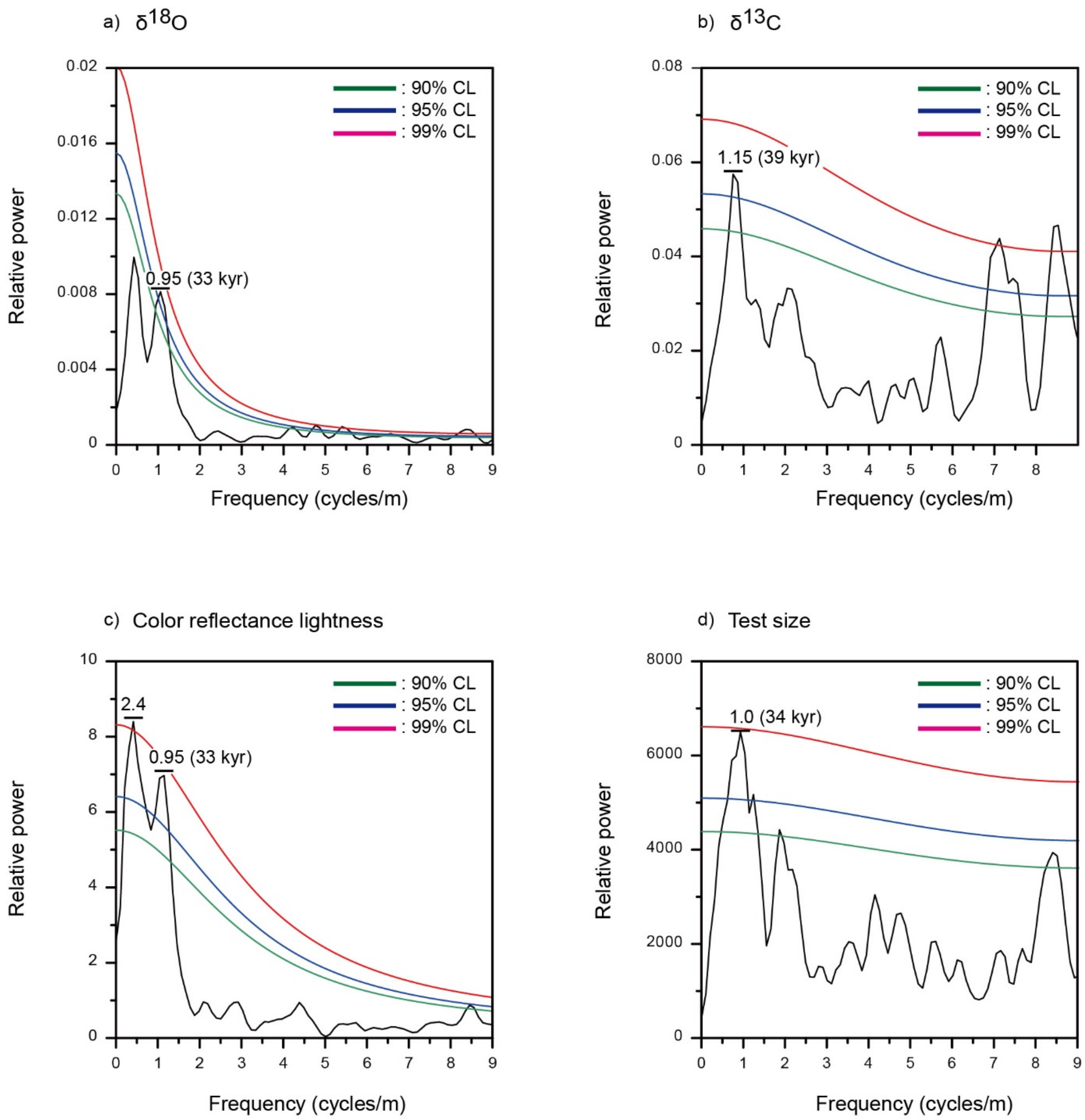

Figure S2. Results of the spectral analysis of proxy records conducted in this study. The analysis was performed using the open-source software REDFIT [7]. The records show periodicities similar to obliquity-related periods (29–55 kyr) during the study interval. CL, confidence level

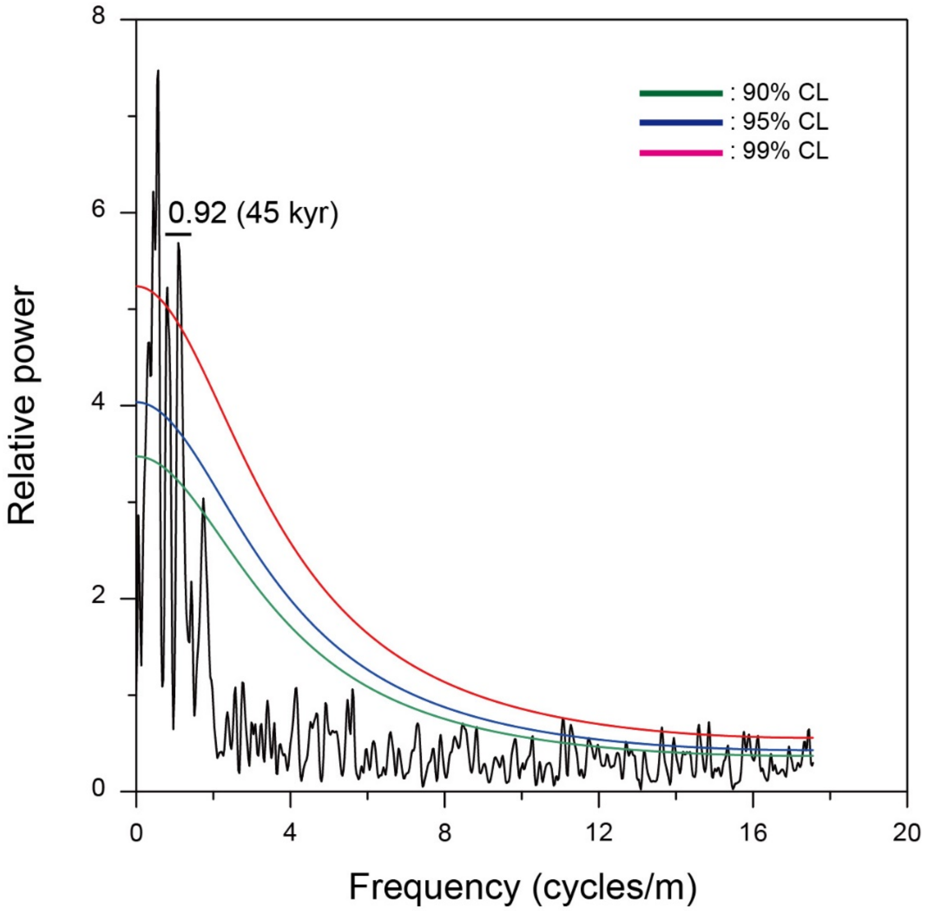

Figure S3. Results of the spectral analysis of changes in color reflectance data (L*) during the entire Late Oligocene Warming (LOW) period using a more robust statistical method for the age model, controlled by 12 magnetic polarity reversals [5, 8]. This analysis was performed using the open-source software REDFIT [7]. A frequency of 0.92 m (45 kyr), almost identical to that of L* (0.95 m) in this study, is seen at the 99% confidence level (CL). This periodicity is included in the obliquity-related period of 29–55 kyrs [9], similar to the periodicities of the proxy records analyzed in this study.

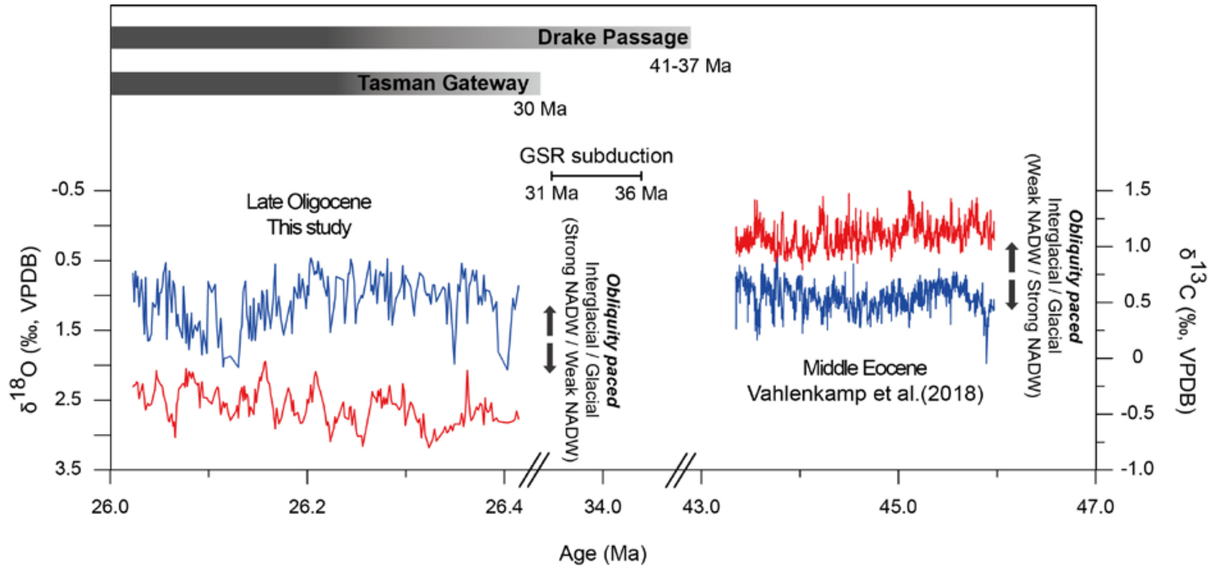

Figure S4. Tectonic evolution with the stable isotope records during the Paleogene. Drake Passage and Tasman Gateway have been known to be opened at 41-37 Ma and 30 Ma, respectively [10, 11, 12, 13, 14]. Also, Greenland-Scotland Ridge (GSR) subduction had started at 36 Ma [15]. The responses of North Atlantic Deep Water (NADW) to the obliquity cycles changed in opposite ways after these tectonic evolution. The red lines and blue lines are oxygen and carbon isotope records, respectively.

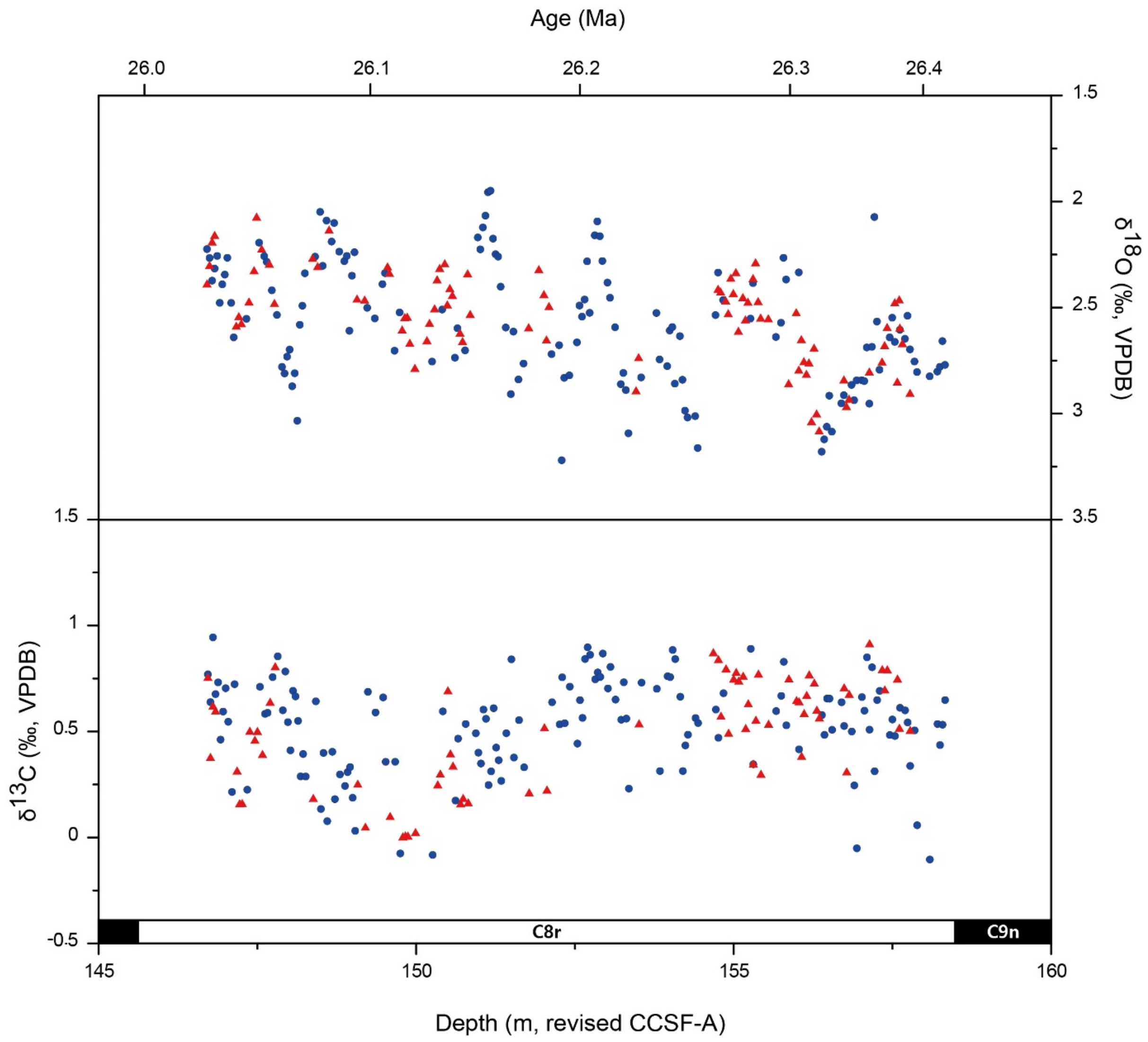

Figure S5. The adjusted interspecies $\delta^{18}O$ (upper) and $\delta^{13}C$ (lower) records. The similar values and trends indicate that the interspecies adjustment performed in this study was successful. Red triangles indicate the $\delta^{18}O$ and $\delta^{13}C$ data of *Cibicidoides* spp., and blue circles indicate those of *O. umbonatus*.

Table S1. Datums used in the age model of this study

Data type	Revised composite depth (m)	Boundary	Age (Ma)	Reference
Paleomagnetostratigraphic	145.22	C8n.2n/C8r	25.987	[5]
Biostratigraphic	155.89	LAD* of *Areoligera semicirculata* and *Saturnodinium pansum* dinocysts	26.300	[6]
Paleomagnetostratigraphic	158.45	C8r/C9n	26.420	[5]
Biostratigraphic	165.59	LAD* of *Enneadocysta pectiniformis* dinocysts	26.700	[6]

*Last appearance datum

Table S2. Oxygen isotope data of *Cibicidoides* spp. and *Oridorsalis umbonatus*. The $\delta^{18}O$ values of *Cibicidoides* spp. were adjusted by adding 0.64‰, and the $\delta^{18}O$ values of *O. umbonatus* were calibrated to the adjusted $\delta^{18}O$ records of *Cibicidoides* spp. by adding 0.14‰ [16, 17]. Data referred to in the main text are the integrated $\delta^{18}O$ values, which include the interspecies average of the adjusted $\delta^{18}O$ values.

**Sample Number**	**Revised composite depth (m)**	**Age (Ma)**	**$\delta^{18}O$ of *Cibicidoides* spp.**	**$\delta^{18}O$ of *Oridorsalis umbonatus***	**Adjusted $\delta^{18}O$ of *Cibicidoides* spp. (+0.64‰)**	**Adjusted $\delta^{18}O$ of *Oridorsalis umbonatus* (+0.14‰)**	**Integrated $\delta^{18}O$**
U1406-2451	146.72	26.023	1.75	2.08	2.39	2.22	2.31
U1406-2453	146.76	26.024	1.66	2.13	2.30	2.27	2.28
U1406-2455	146.8	26.026	1.55	2.23	2.19	2.37	2.28
U1406-2457	146.84	26.027	1.52	2.18	2.16	2.32	2.24
U1406-2458	146.88	26.028		2.12		2.26	2.26
U1406-2459	146.92	26.029		2.34		2.48	2.48
U1406-2460	146.96	26.030		2.25		2.39	2.39
U1406-2461	147	26.032		2.20		2.34	2.34
U1406-2462	147.04	26.033		2.12		2.26	2.26
U1406-2463	147.1	26.035		2.34		2.48	2.48
U1406-2464	147.14	26.036		2.50		2.64	2.64
U1406-2465	147.18	26.037	1.95		2.59		2.59
U1406-2466	147.22	26.038	1.91		2.55		2.55
U1406-2467	147.26	26.040	1.94		2.58		2.58
U1406-2469	147.34	26.042		2.41		2.55	2.55
U1406-2470	147.38	26.043	1.84		2.48		2.48
U1406-2472	147.46	26.046	1.69		2.33		2.33
U1406-2473	147.5	26.047	1.44		2.08		2.08
U1406-2474	147.54	26.048		2.05		2.19	2.19
U1406-2475	147.58	26.049	1.59		2.23		2.23
U1406-2476	147.62	26.050		2.12		2.26	2.26
U1406-2477	147.66	26.052		2.14		2.28	2.28
U1406-2478	147.7	26.053	1.66		2.30		2.30

Sample Number	Revised composite depth (m)	Age (Ma)	$\delta^{18}O$ of *Cibicidoides* spp.	$\delta^{18}O$ of *Oridorsalis umbonatus*	Adjusted $\delta^{18}O$ of *Cibicidoides* spp. (+0.64‰)	Adjusted $\delta^{18}O$ of *Oridorsalis umbonatus* (+0.14‰)	Integrated $\delta^{18}O$
U1406-2479	147.74	26.054	;	2.28		2.42	2.42
U1406-2480	147.78	26.055	1.84		2.48		2.48
U1406-2481	147.82	26.056		2.39		2.53	2.53
U1406-2483	147.9	26.059		2.64		2.78	2.78
U1406-2484	147.94	26.060		2.67		2.81	2.81
U1406-2485	147.98	26.061		2.59		2.73	2.73
U1406-2486	148.02	26.062		2.56		2.70	2.70
U1406-2487	148.06	26.064		2.73		2.87	2.87
U1406-2488	148.1	26.065		2.67		2.81	2.81
U1406-2489	148.14	26.066		2.89		3.03	3.03
U1406-2490	148.18	26.067		2.44		2.58	2.58
U1406-2491	148.22	26.068		2.35		2.49	2.49
U1406-2492	148.26	26.070		2.20		2.34	2.34
U1406-2495	148.38	26.073	1.63		2.27		2.27
U1406-2496	148.42	26.075		2.12		2.26	2.26
U1406-2497	148.46	26.076	1.67		2.31		2.31
U1406-2498	148.5	26.077		1.91		2.05	2.05
U1406-2499	148.54	26.078		2.16		2.30	2.30
U1406-2500	148.6	26.080		1.95		2.09	2.09
U1406-2501	148.64	26.081	1.50		2.14		2.14
U1406-2502	148.68	26.082		2.05		2.19	2.19
U1406-2503	148.72	26.084		1.96		2.10	2.10
U1406-2505	148.8	26.086		2.10		2.24	2.24
U1406-2507	148.88	26.088		2.14		2.28	2.28
U1406-2508	148.92	26.090		2.12		2.26	2.26
U1406-2509	148.96	26.091		2.47		2.61	2.61

**Sample Number**	**Revised composite depth (m)**	**Age (Ma)**	**δ18O of *Cibicidoides* spp.**	**δ18O of *Oridorsalis umbonatus***	**Adjusted δ18O of *Cibicidoides* spp. (+0.64‰)**	**Adjusted δ18O of *Oridorsalis umbonatus* (+0.14‰)**	**Integrated δ18O**
U1406-2510	149	26.092		2.21		2.35	2.35
U1406-2511	149.04	26.093		2.10		2.24	2.24
U1406-2512	149.08	26.094	1.82		2.46		2.46
U1406-2515	149.2	26.098	1.83		2.47		2.47
U1406-2516	149.24	26.099		2.36		2.50	2.50
U1406-2519	149.36	26.103		2.41		2.55	2.55
U1406-2524	149.48	26.107		2.25		2.39	2.39
U1406-2526	149.52	26.108		2.20		2.34	2.34
U1406-2528	149.56	26.109	1.67		2.31		2.31
U1406-2529	149.59	26.110	1.70		2.34		2.34
U1406-2533	149.67	26.112		2.56		2.70	2.70
U1406-2536	149.75	26.115		2.38		2.52	2.52
U1406-2537	149.79	26.116	1.97		2.61		2.61
U1406-2538	149.83	26.117	1.91		2.55		2.55
U1406-2539	149.87	26.118	1.91		2.55		2.55
U1406-2540	149.91	26.120	2.03		2.67		2.67
U1406-2542	149.99	26.122	2.15		2.79		2.79
U1406-2546	150.18	26.128	2.02		2.66		2.66
U1406-2547	150.22	26.129	1.94		2.58		2.58
U1406-2548	150.26	26.130		2.61		2.75	2.75
U1406-2549	150.3	26.131	1.87		2.51		2.51
U1406-2550	150.34	26.132	1.73		2.37		2.37
U1406-2551	150.38	26.134	1.68		2.32		2.32
U1406-2552	150.42	26.135		2.37		2.51	2.51
U1406-2553	150.46	26.136	1.66		2.30		2.30
U1406-2554	150.5	26.137	1.85		2.49		2.49

Sample Number	Revised composite depth (m)	Age (Ma)	$\delta^{18}O$ of *Cibicidoides* spp.	$\delta^{18}O$ of *Oridorsalis umbonatus*	Adjusted $\delta^{18}O$ of *Cibicidoides* spp. (+0.64‰)	Adjusted $\delta^{18}O$ of *Oridorsalis umbonatus* (+0.14‰)	Integrated $\delta^{18}O$
U1406-2555	150.54	26.139	1.77		2.41		2.41
U1406-2556	150.58	26.140	1.81		2.45		2.45
U1406-2557	150.62	26.141		2.60		2.74	2.74
U1406-2558	150.66	26.142		2.46		2.60	2.60
U1406-2559	150.7	26.143	1.98		2.62		2.62
U1406-2560	150.74	26.145	2.02		2.66		2.66
U1406-2561	150.78	26.146		2.56		2.70	2.70
U1406-2562	150.82	26.147	1.70		2.34		2.34
U1406-2563	150.86	26.148	1.90		2.54		2.54
U1406-2565	150.94	26.151		2.00		2.14	2.14
U1406-2566	150.98	26.152		2.03		2.17	2.17
U1406-2567	151.02	26.153		2.08		2.22	2.22
U1406-2568	151.06	26.154		1.98		2.12	2.12
U1406-2569	151.1	26.155		1.93		2.07	2.07
U1406-2570	151.14	26.157		1.82		1.96	1.96
U1406-2571	151.18	26.158		1.81		1.95	1.95
U1406-2572	151.22	26.159		2.03		2.17	2.17
U1406-2573	151.26	26.160		2.11		2.25	2.25
U1406-2574	151.3	26.161		2.12		2.26	2.26
U1406-2575	151.34	26.163		2.26		2.40	2.40
U1406-2577	151.42	26.165		2.45		2.59	2.59
U1406-2579	151.5	26.167		2.77		2.91	2.91
U1406-2580	151.54	26.169		2.47		2.61	2.61
U1406-2582	151.62	26.171		2.70		2.84	2.84
U1406-2583	151.7	26.174		2.62		2.76	2.76
U1406-2585	151.78	26.176	1.96		2.60		2.60

Sample Number	Revised composite depth (m)	Age (Ma)	$\delta^{18}O$ of *Cibicidoides* spp.	$\delta^{18}O$ of *Oridorsalis umbonatus*	Adjusted $\delta^{18}O$ of *Cibicidoides* spp. (+0.64‰)	Adjusted $\delta^{18}O$ of *Oridorsalis umbonatus* (+0.14‰)	Integrated $\delta^{18}O$
U1406-2589	151.94	26.181	1.68		2.32		2.32
U1406-2591	152.02	26.183	1.80		2.44		2.44
U1406-2592	152.06	26.184	2.02		2.66		2.66
U1406-2593	152.1	26.186	1.86		2.50		2.50
U1406-2594	152.14	26.187		2.58		2.72	2.72
U1406-2597	152.26	26.190		2.54		2.68	2.68
U1406-2599	152.34	26.193		2.69		2.83	2.83
U1406-2601	152.42	26.195		2.68		2.82	2.82
U1406-2604	152.54	26.199		2.52		2.66	2.66
U1406-2605	152.58	26.200		2.35		2.49	2.49
U1406-2606	152.62	26.201		2.40		2.54	2.54
U1406-2607	152.66	26.203		2.32		2.46	2.46
U1406-2608	152.7	26.204		2.14		2.28	2.28
U1406-2609	152.74	26.205		2.38		2.52	2.52
U1406-2611	152.82	26.207		2.02		2.16	2.16
U1406-2612	152.86	26.209		1.95		2.09	2.09
U1406-2613	152.9	26.210		2.02		2.16	2.16
U1406-2614	152.94	26.211		2.14		2.28	2.28
U1406-2616	153.02	26.213		2.24		2.38	2.38
U1406-2617	153.06	26.215		2.31		2.45	2.45
U1406-2619	153.14	26.217		2.45		2.59	2.59
U1406-2620	153.23	26.220		2.72		2.86	2.86
U1406-2621	153.27	26.221		2.67		2.81	2.81
U1406-2622	153.31	26.222		2.75		2.89	2.89
U1406-2623	153.35	26.223		2.95		3.09	3.09
U1406-2626	153.47	26.227	2.26		2.90		2.90

Sample Number	Revised composite depth (m)	Age (Ma)	$\delta^{18}O$ of *Cibicidoides* spp.	$\delta^{18}O$ of *Oridorsalis umbonatus*	Adjusted $\delta^{18}O$ of *Cibicidoides* spp. (+0.64‰)	Adjusted $\delta^{18}O$ of *Oridorsalis umbonatus* (+0.14‰)	Integrated $\delta^{18}O$
U1406-2627	153.51	26.228	2.10		2.74		2.74
U1406-2628	153.55	26.229		2.69		2.83	2.83
U1406-2638	153.79	26.237		2.39		2.53	2.53
U1406-2641	153.84	26.238		2.60		2.74	2.74
U1406-2647	153.96	26.242		2.64		2.78	2.78
U1406-2648	154	26.243		2.47		2.61	2.61
U1406-2649	154.04	26.244		2.45		2.59	2.59
U1406-2650	154.08	26.245		2.72		2.86	2.86
U1406-2652	154.16	26.248		2.49		2.63	2.63
U1406-2653	154.2	26.249		2.70		2.84	2.84
U1406-2654	154.24	26.250		2.85		2.99	2.99
U1406-2655	154.28	26.251		2.88		3.02	3.02
U1406-2658	154.4	26.255		2.87		3.01	3.01
U1406-2659	154.44	26.256		3.02		3.16	3.16
U1406-2666	154.72	26.265		2.39		2.53	2.53
U1406-2667	154.76	26.266	1.78	2.19	2.42	2.33	2.38
U1406-2668	154.8	26.267	1.79		2.43		2.43
U1406-2669	154.84	26.268		2.32		2.46	2.46
U1406-2670	154.88	26.270	1.83		2.47		2.47
U1406-2671	154.92	26.271	1.89		2.53		2.53
U1406-2672	154.96	26.272	1.72		2.36		2.36
U1406-2673	155	26.273	1.80		2.44		2.44
U1406-2674	155.04	26.274	1.70		2.34		2.34
U1406-2675	155.08	26.276	1.97		2.61		2.61
U1406-2676	155.15	26.278	1.82		2.46		2.46
U1406-2677	155.19	26.279	1.92		2.56		2.56

Sample Number	Revised composite depth (m)	Age (Ma)	$\delta^{18}O$ of *Cibicidoides* spp.	$\delta^{18}O$ of *Oridorsalis umbonatus*	Adjusted $\delta^{18}O$ of *Cibicidoides* spp. (+0.64‰)	Adjusted $\delta^{18}O$ of *Oridorsalis umbonatus* (+0.14‰)	Integrated $\delta^{18}O$
U1406-2678	155.23	26.280	1.84		2.48		2.48
U1406-2679	155.27	26.281		2.41		2.55	2.55
U1406-2680	155.31	26.282	1.73	2.24	2.37	2.38	2.38
U1406-2681	155.35	26.284	1.65		2.29		2.29
U1406-2682	155.39	26.285	1.83		2.47		2.47
U1406-2683	155.43	26.286	1.91		2.55		2.55
U1406-2686	155.55	26.290	1.92		2.56		2.56
U1406-2689	155.67	26.293		2.50		2.64	2.64
U1406-2691	155.75	26.296		2.43		2.57	2.57
U1406-2692	155.79	26.297		2.12		2.26	2.26
U1406-2693	155.83	26.298		2.23		2.37	2.37
U1406-2694	155.87	26.299	2.22		2.86		2.86
U1406-2697	155.99	26.305	1.89		2.53		2.53
U1406-2698	156.03	26.307	2.16	2.19	2.80	2.33	2.57
U1406-2699	156.07	26.308	2.01		2.65		2.65
U1406-2700	156.11	26.310	2.12		2.76		2.76
U1406-2701	156.15	26.312	2.18		2.82		2.82
U1406-2702	156.19	26.314	2.12		2.76		2.76
U1406-2703	156.23	26.316	2.40		3.04		3.04
U1406-2704	156.27	26.318	2.05		2.69		2.69
U1406-2705	156.31	26.320	2.36		3.00		3.00
U1406-2706	156.35	26.322	2.44		3.08		3.08
U1406-2707	156.39	26.323		3.04		3.18	3.18
U1406-2708	156.43	26.325		2.98		3.12	3.12
U1406-2709	156.47	26.327		2.92		3.06	3.06
U1406-2710	156.51	26.329		2.77		2.91	2.91

Sample Number	Revised composite depth (m)	Age (Ma)	$\delta^{18}O$ of *Cibicidoides* spp.	$\delta^{18}O$ of *Oridorsalis umbonatus*	Adjusted $\delta^{18}O$ of *Cibicidoides* spp. (+0.64‰)	Adjusted $\delta^{18}O$ of *Oridorsalis umbonatus* (+0.14‰)	Integrated $\delta^{18}O$
U1406-2711	156.55	26.331		2.94		3.08	3.08
U1406-2714	156.7	26.338		2.81		2.95	2.95
U1406-2715	156.74	26.340	2.20	2.77	2.84	2.91	2.88
U1406-2716	156.78	26.342	2.33		2.97		2.97
U1406-2717	156.82	26.344	2.29		2.93		2.93
U1406-2718	156.86	26.345		2.72		2.86	2.86
U1406-2719	156.9	26.347		2.80		2.94	2.94
U1406-2720	156.94	26.349		2.70		2.84	2.84
U1406-2722	157.02	26.353		2.70		2.84	2.84
U1406-2723	157.06	26.355		2.71		2.85	2.85
U1406-2724	157.1	26.357		2.55		2.69	2.69
U1406-2725	157.14	26.359	2.17	2.81	2.81	2.95	2.88
U1406-2726	157.18	26.360		2.54		2.68	2.68
U1406-2727	157.22	26.362		1.93		2.07	2.07
U1406-2728	157.26	26.364		2.43		2.57	2.57
U1406-2729	157.3	26.366		2.65		2.79	2.79
U1406-2730	157.34	26.368	2.12		2.76		2.76
U1406-2731	157.38	26.370	2.04		2.68		2.68
U1406-2732	157.42	26.372	1.96		2.60		2.60
U1406-2733	157.46	26.374		2.50		2.64	2.64
U1406-2734	157.5	26.375		2.41		2.55	2.55
U1406-2735	157.54	26.377	1.84	2.52	2.48	2.66	2.57
U1406-2736	157.58	26.379	2.21		2.85		2.85
U1406-2737	157.61	26.381	1.83		2.47		2.47
U1406-2738	157.62	26.381	1.96	2.46	2.60	2.60	2.60
U1406-2740	157.66	26.383	2.03		2.67		2.67

**Sample Number**	**Revised composite depth (m)**	**Age (Ma)**	**δ^{18}O of *Cibicidoides* spp.**	**δ^{18}O of *Oridorsalis umbonatus***	**Adjusted δ^{18}O of *Cibicidoides* spp. (+0.64‰)**	**Adjusted δ^{18}O of *Oridorsalis umbonatus* (+0.14‰)**	**Integrated δ^{18}O**
U1406-2742	157.7	26.385		2.51		2.65	2.65
U1406-2744	157.74	26.387		2.40		2.54	2.54
U1406-2746	157.78	26.389	2.27	2.56	2.91	2.70	2.80
U1406-2749	157.85	26.392		2.61		2.75	2.75
U1406-2751	157.89	26.394		2.66		2.80	2.80
U1406-2758	158.09	26.403		2.68		2.82	2.82
U1406-2761	158.21	26.409		2.66		2.80	2.80
U1406-2762	158.25	26.411		2.64		2.78	2.78
U1406-2763	158.29	26.412		2.52		2.66	2.66
U1406-2764	158.33	26.414		2.63		2.77	2.77

Table S3. Carbon isotope data of *Cibicidoides* spp. and *Oridorsalis umbonatus*. The $\delta^{13}C$ values of *O. umbonatus* were adjusted to those of *Cibicidoides* spp. by adding 1.19‰ [16, 17]. Data referred to in the main text are the integrated $\delta^{13}C$ values, which include the interspecies average of the adjusted $\delta^{13}C$ values.

**Sample Number**	**Revised composite depth (m)**	**Age (Ma)**	**$\delta^{13}C$ of *Cibicidoides* spp.**	**$\delta^{13}C$ of *Oridorsalis umbonatus***	**Adjusted $\delta^{13}C$ of *Cibicidoides* spp.**	**Adjusted $\delta^{13}C$ of *Oridorsalis umbonatus* (+1.19‰)**	**Integrated $\delta^{13}C$**
U1406-2451	146.72	26.023	0.75	-0.42	0.75	0.77	0.76
U1406-2453	146.76	26.024	0.37	-0.55	0.37	0.64	0.51
U1406-2455	146.8	26.026	0.62	-0.25	0.62	0.94	0.78
U1406-2457	146.84	26.027	0.59	-0.51	0.59	0.68	0.64
U1406-2458	146.88	26.028		-0.46		0.73	0.73
U1406-2459	146.92	26.029		-0.73		0.46	0.46
U1406-2460	146.96	26.030		-0.60		0.59	0.59
U1406-2461	147	26.032		-0.49		0.70	0.70
U1406-2462	147.04	26.033		-0.64		0.55	0.55
U1406-2463	147.1	26.035		-0.97		0.22	0.22
U1406-2464	147.14	26.036		-0.47		0.72	0.72
U1406-2465	147.18	26.037	0.31		0.31		0.31
U1406-2466	147.22	26.038	0.16		0.16		0.16
U1406-2467	147.26	26.040	0.16		0.16		0.16
U1406-2469	147.34	26.042		-0.96		0.23	0.23
U1406-2470	147.38	26.043	0.50		0.50		0.50
U1406-2472	147.46	26.046	0.46		0.46		0.46
U1406-2473	147.5	26.047	0.50		0.50		0.50
U1406-2474	147.54	26.048		-0.48		0.71	0.71
U1406-2475	147.58	26.049	0.39		0.39		0.39
U1406-2476	147.62	26.050		-0.61		0.58	0.58
U1406-2477	147.66	26.052		-0.60		0.59	0.59
U1406-2478	147.7	26.053	0.64		0.64		0.64

Sample Number	Revised composite depth (m)	Age (Ma)	δ^{13}C of *Cibicidoides* spp.	δ^{13}C of *Oridorsalis umbonatus*	Adjusted δ^{13}C of *Cibicidoides* spp.	Adjusted δ^{13}C of *Oridorsalis umbonatus* (+1.19‰)	Integrated δ^{13}C
U1406-2479	147.74	26.054		-0.43		0.76	0.76
U1406-2480	147.78	26.055	0.80		0.80		0.80
U1406-2481	147.82	26.056		-0.33		0.86	0.86
U1406-2482	147.86	26.058		-1.02		0.17	0.17
U1406-2483	147.9	26.059		-0.59		0.60	0.60
U1406-2484	147.94	26.060		-0.41		0.78	0.78
U1406-2485	147.98	26.061		-0.65		0.54	0.54
U1406-2486	148.02	26.062		-0.78		0.41	0.41
U1406-2487	148.06	26.064		-0.50		0.69	0.69
U1406-2488	148.1	26.065		-0.52		0.67	0.67
U1406-2489	148.14	26.066		-0.64		0.55	0.55
U1406-2490	148.18	26.067		-0.90		0.29	0.29
U1406-2491	148.22	26.068		-0.80		0.39	0.39
U1406-2492	148.26	26.070		-0.90		0.29	0.29
U1406-2495	148.38	26.073	0.18		0.18		0.18
U1406-2496	148.42	26.075		-0.55		0.64	0.64
U1406-2498	148.5	26.077		-1.05		0.14	0.14
U1406-2499	148.54	26.078		-0.79		0.40	0.40
U1406-2500	148.6	26.080		-1.11		0.08	0.08
U1406-2502	148.68	26.082		-0.79		0.40	0.40
U1406-2503	148.72	26.084		-1.01		0.18	0.18
U1406-2505	148.8	26.086		-0.89		0.30	0.30
U1406-2507	148.88	26.088		-0.95		0.24	0.24
U1406-2508	148.92	26.090		-0.88		0.31	0.31
U1406-2509	148.96	26.091		-0.86		0.33	0.33
U1406-2510	149	26.092		-1.00		0.19	0.19

Sample Number	Revised composite depth (m)	Age (Ma)	δ13C of *Cibicidoides* spp.	δ13C of *Oridorsalis umbonatus*	Adjusted δ13C of *Cibicidoides* spp.	Adjusted δ13C of *Oridorsalis umbonatus* (+1.19‰)	Integrated δ13C
U1406-2511	149.04	26.093		-1.16		0.03	0.03
U1406-2512	149.08	26.094	0.25		0.25		0.25
U1406-2515	149.2	26.098	0.05		0.05		0.05
U1406-2516	149.24	26.099		-0.50		0.69	0.69
U1406-2519	149.36	26.103		-0.60		0.59	0.59
U1406-2524	149.48	26.107		-0.53		0.66	0.66
U1406-2526	149.52	26.108		-0.83		0.36	0.36
U1406-2529	149.59	26.110	0.10		0.10		0.10
U1406-2533	149.67	26.112		-0.83		0.36	0.36
U1406-2536	149.75	26.115		-1.26		-0.07	-0.07
U1406-2537	149.79	26.116	0.00		0.00		0.00
U1406-2538	149.83	26.117	0.00		0.00		0.00
U1406-2539	149.87	26.118	0.00		0.00		0.00
U1406-2542	149.99	26.122	0.02		0.02		0.02
U1406-2548	150.26	26.130		-1.27		-0.08	-0.08
U1406-2550	150.34	26.132	0.25		0.25		0.25
U1406-2551	150.38	26.134	0.30		0.30		0.30
U1406-2552	150.42	26.135		-0.59		0.60	0.60
U1406-2554	150.5	26.137	0.69		0.69		0.69
U1406-2555	150.54	26.139	0.39		0.39		0.39
U1406-2556	150.58	26.140	0.33		0.33		0.33
U1406-2557	150.62	26.141		-1.02		0.17	0.17
U1406-2558	150.66	26.142		-0.72		0.47	0.47
U1406-2559	150.7	26.143	0.16		0.16		0.16
U1406-2560	150.74	26.145	0.18		0.18		0.18
U1406-2561	150.78	26.146		-0.65		0.54	0.54

**Sample Number**	**Revised composite depth (m)**	**Age (Ma)**	**δ^{13}C of *Cibicidoides* spp.**	**δ^{13}C of *Oridorsalis umbonatus***	**Adjusted δ^{13}C of *Cibicidoides* spp.**	**Adjusted δ^{13}C of *Oridorsalis umbonatus* (+1.19‰)**	**Integrated δ^{13}C**
U1406-2562	150.82	26.147	0.16		0.16		0.16
U1406-2565	150.94	26.151		-0.70		0.49	0.49
U1406-2566	150.98	26.152		-0.79		0.40	0.40
U1406-2567	151.02	26.153		-0.84		0.35	0.35
U1406-2568	151.06	26.154		-0.59		0.60	0.60
U1406-2569	151.1	26.155		-0.63		0.56	0.56
U1406-2570	151.14	26.157		-0.94		0.25	0.25
U1406-2571	151.18	26.158		-0.88		0.31	0.31
U1406-2572	151.22	26.159		-0.58		0.61	0.61
U1406-2573	151.26	26.160		-0.77		0.42	0.42
U1406-2574	151.3	26.161		-0.83		0.36	0.36
U1406-2575	151.34	26.163		-0.92		0.27	0.27
U1406-2577	151.42	26.165		-0.70		0.49	0.49
U1406-2579	151.5	26.167		-0.35		0.84	0.84
U1406-2580	151.54	26.169		-0.81		0.38	0.38
U1406-2582	151.62	26.171		-0.64		0.55	0.55
U1406-2583	151.7	26.174		-0.86		0.33	0.33
U1406-2585	151.78	26.176	0.21		0.21		0.21
U1406-2591	152.02	26.183	0.51		0.51		0.51
U1406-2592	152.06	26.184	0.22		0.22		0.22
U1406-2594	152.14	26.187		-0.55		0.64	0.64
U1406-2597	152.26	26.190		-0.66		0.53	0.53
U1406-2598	152.3	26.192		-0.43		0.76	0.76
U1406-2599	152.34	26.193		-0.65		0.54	0.54
U1406-2601	152.42	26.195		-0.48		0.71	0.71
U1406-2604	152.54	26.199		-0.75		0.44	0.44

Sample Number	Revised composite depth (m)	Age (Ma)	δ^{13}C of *Cibicidoides* spp.	δ^{13}C of *Oridorsalis umbonatus*	Adjusted δ^{13}C of *Cibicidoides* spp.	Adjusted δ^{13}C of *Oridorsalis umbonatus* (+1.19‰)	Integrated δ^{13}C
U1406-2605	152.58	26.200		-0.54		0.65	0.65
U1406-2606	152.62	26.201		-0.63		0.56	0.56
U1406-2607	152.66	26.203		-0.35		0.84	0.84
U1406-2608	152.7	26.204		-0.29		0.90	0.90
U1406-2609	152.74	26.205		-0.33		0.86	0.86
U1406-2611	152.82	26.207		-0.44		0.75	0.75
U1406-2612	152.86	26.209		-0.41		0.78	0.78
U1406-2613	152.9	26.210		-0.43		0.76	0.76
U1406-2614	152.94	26.211		-0.32		0.87	0.87
U1406-2616	153.02	26.213		-0.49		0.70	0.70
U1406-2617	153.06	26.215		-0.38		0.81	0.81
U1406-2619	153.14	26.217		-0.54		0.65	0.65
U1406-2620	153.23	26.220		-0.63		0.56	0.56
U1406-2621	153.27	26.221		-0.46		0.73	0.73
U1406-2622	153.31	26.222		-0.63		0.56	0.56
U1406-2623	153.35	26.223		-0.96		0.23	0.23
U1406-2627	153.51	26.228	0.53		0.53		0.53
U1406-2628	153.55	26.229		-0.46		0.73	0.73
U1406-2638	153.79	26.237		-0.49		0.70	0.70
U1406-2641	153.84	26.238		-0.88		0.31	0.31
U1406-2647	153.96	26.242		-0.43		0.76	0.76
U1406-2648	154	26.243		-0.43		0.76	0.76
U1406-2649	154.04	26.244		-0.30		0.89	0.89
U1406-2650	154.08	26.245		-0.35		0.84	0.84
U1406-2652	154.16	26.248		-0.53		0.66	0.66
U1406-2653	154.2	26.249		-0.88		0.31	0.31

Sample Number	Revised composite depth (m)	Age (Ma)	$\delta^{13}C$ of *Cibicidoides* spp.	$\delta^{13}C$ of *Oridorsalis umbonatus*	Adjusted $\delta^{13}C$ of *Cibicidoides* spp.	Adjusted $\delta^{13}C$ of *Oridorsalis umbonatus* (+1.19‰)	Integrated $\delta^{13}C$
U1406-2654	154.24	26.250		-0.76		0.43	0.43
U1406-2655	154.28	26.251		-0.70		0.49	0.49
U1406-2658	154.4	26.255		-0.63		0.56	0.56
U1406-2659	154.44	26.256		-0.65		0.54	0.54
U1406-2665	154.68	26.263	0.87		0.87		0.87
U1406-2666	154.72	26.265		-0.59		0.60	0.60
U1406-2667	154.76	26.266	0.84	-0.72	0.84	0.47	0.65
U1406-2668	154.8	26.267	0.57		0.57		0.57
U1406-2669	154.84	26.268		-0.51		0.68	0.68
U1406-2670	154.88	26.270	0.79		0.79		0.79
U1406-2671	154.92	26.271	0.49		0.49		0.49
U1406-2673	155	26.273	0.74		0.74		0.74
U1406-2674	155.04	26.274	0.78		0.78		0.78
U1406-2675	155.08	26.276	0.74		0.74		0.74
U1406-2676	155.15	26.278	0.76		0.76		0.76
U1406-2677	155.19	26.279	0.51		0.51		0.51
U1406-2678	155.23	26.280	0.63		0.63		0.63
U1406-2679	155.27	26.281		-0.30		0.89	0.89
U1406-2680	155.31	26.282	0.34	-0.84	0.34	0.35	0.34
U1406-2681	155.35	26.284	0.55		0.55		0.55
U1406-2682	155.39	26.285	0.77		0.77		0.77
U1406-2683	155.43	26.286	0.30		0.30		0.30
U1406-2686	155.55	26.290	0.53		0.53		0.53
U1406-2689	155.67	26.293		-0.59		0.60	0.60
U1406-2691	155.75	26.296		-0.52		0.67	0.67
U1406-2692	155.79	26.297		-0.36		0.83	0.83

Sample Number	Revised composite depth (m)	Age (Ma)	δ^{13}C of *Cibicidoides* spp.	δ^{13}C of *Oridorsalis umbonatus*	Adjusted δ^{13}C of *Cibicidoides* spp.	Adjusted δ^{13}C of *Oridorsalis umbonatus* (+1.19‰)	Integrated δ^{13}C
U1406-2693	155.83	26.298		-0.66		0.53	0.53
U1406-2694	155.87	26.299	0.75		0.75		0.75
U1406-2697	155.99	26.305	0.64		0.64		0.64
U1406-2698	156.03	26.307	0.64	-0.77	0.64	0.42	0.53
U1406-2699	156.07	26.308	0.38		0.38		0.38
U1406-2700	156.11	26.310	0.58		0.58		0.58
U1406-2701	156.15	26.312	0.67		0.67		0.67
U1406-2702	156.19	26.314	0.76		0.76		0.76
U1406-2704	156.27	26.318	0.73		0.73		0.73
U1406-2705	156.31	26.320	0.60		0.60		0.60
U1406-2706	156.35	26.322	0.56		0.56		0.56
U1406-2707	156.39	26.323		-0.61		0.58	0.58
U1406-2708	156.43	26.325		-0.71		0.48	0.48
U1406-2709	156.47	26.327		-0.53		0.66	0.66
U1406-2710	156.51	26.329		-0.53		0.66	0.66
U1406-2711	156.55	26.331		-0.68		0.51	0.51
U1406-2714	156.7	26.338		-0.55		0.64	0.64
U1406-2715	156.74	26.340	0.70	-0.66	0.70	0.53	0.61
U1406-2716	156.78	26.342	0.31		0.31		0.31
U1406-2717	156.82	26.344	0.67		0.67		0.67
U1406-2718	156.86	26.345		-0.69		0.50	0.50
U1406-2719	156.9	26.347		-0.94		0.25	0.25
U1406-2720	156.94	26.349		-1.24		-0.05	-0.05
U1406-2722	157.02	26.353		-0.53		0.66	0.66
U1406-2723	157.06	26.355		-0.59		0.60	0.60
U1406-2724	157.1	26.357		-0.34		0.85	0.85

Sample Number	Revised composite depth (m)	Age (Ma)	$\delta^{13}C$ of *Cibicidoides* spp.	$\delta^{13}C$ of *Oridorsalis umbonatus*	Adjusted $\delta^{13}C$ of *Cibicidoides* spp.	Adjusted $\delta^{13}C$ of *Oridorsalis umbonatus* (+1.19‰)	Integrated $\delta^{13}C$
U1406-2725	157.14	26.359	0.91	-0.68	0.91	0.51	0.71
U1406-2726	157.18	26.360		-0.39		0.80	0.80
U1406-2727	157.22	26.362		-0.88		0.31	0.31
U1406-2728	157.26	26.364		-0.54		0.65	0.65
U1406-2729	157.3	26.366		-0.50		0.69	0.69
U1406-2730	157.34	26.368	0.79		0.79		0.79
U1406-2731	157.38	26.370	0.69		0.69		0.69
U1406-2732	157.42	26.372	0.79		0.79		0.79
U1406-2733	157.46	26.374		-0.71		0.48	0.48
U1406-2734	157.5	26.375		-0.63		0.56	0.56
U1406-2735	157.54	26.377		-0.71		0.48	0.48
U1406-2736	157.58	26.379	0.74		0.74		0.74
U1406-2737	157.61	26.381	0.51		0.51		0.51
U1406-2738	157.62	26.381		-0.58		0.61	0.61
U1406-2742	157.7	26.385		-0.59		0.60	0.60
U1406-2744	157.74	26.387		-0.65		0.54	0.54
U1406-2746	157.78	26.389	0.50	-0.85	0.50	0.34	0.42
U1406-2749	157.85	26.392		-0.68		0.51	0.51
U1406-2751	157.89	26.394		-1.13		0.06	0.06
U1406-2758	158.09	26.403		-1.29		-0.10	-0.10
U1406-2761	158.21	26.409		-0.65		0.54	0.54
U1406-2762	158.25	26.411		-0.75		0.44	0.44
U1406-2763	158.29	26.412		-0.66		0.53	0.53
U1406-2764	158.33	26.414		-0.54		0.65	0.65

Table S4. Calcium carbonate ($CaCO_3$) contents in this study

**Revised composite depth (m)**	**Age (Ma)**	**$CaCO_3$ (wt%)**
146.72	26.02	39.95
146.80	26.03	46.98
147.04	26.03	33.90
147.22	26.04	37.07
147.38	26.04	17.94
147.54	26.05	41.24
147.70	26.05	39.36
147.86	26.06	27.86
148.02	26.06	33.51
148.18	26.07	26.92
148.34	26.07	38.14
148.50	26.08	45.16
148.68	26.08	38.82
148.84	26.09	41.73
149.00	26.09	30.75
149.16	26.10	35.76
149.32	26.10	23.16
149.48	26.11	31.60
149.63	26.11	35.28
149.79	26.12	29.37
150.11	26.13	28.40
150.30	26.13	38.19
150.46	26.14	43.28
150.62	26.14	48.99
150.78	26.15	40.47
150.94	26.15	45.99
151.10	26.16	38.90
151.26	26.16	37.38
151.42	26.17	39.52
151.58	26.17	43.80
151.78	26.18	41.90
151.94	26.18	48.24
152.10	26.19	48.92
152.26	26.19	39.84
152.46	26.20	22.86
152.58	26.20	35.95
152.74	26.20	51.56
152.90	26.21	45.43
153.06	26.21	48.11
153.27	26.22	44.51
153.51	26.23	33.76
153.67	26.23	28.26
153.79	26.24	44.42
153.84	26.24	45.39
153.92	26.24	41.55
154.44	26.26	28.45
155.19	26.28	46.79
155.31	26.28	38.20
155.43	26.29	35.61
155.63	26.29	40.75
155.75	26.30	47.48
155.87	26.30	42.55

**Revised composite depth (m)**	**Age (Ma)**	**$CaCO_3$ (wt%)**
156.03	26.31	45.40
156.19	26.31	41.22
156.31	26.32	48.06
156.47	26.33	43.97
156.66	26.34	46.27
156.82	26.34	45.16
156.98	26.35	40.99
157.14	26.36	48.30
157.30	26.37	57.13
157.46	26.37	50.64
157.61	26.38	39.05
157.74	26.39	35.30
157.89	26.39	30.41
158.05	26.40	26.05
158.21	26.41	31.58
158.37	26.42	31.45
158.53	26.42	34.46
158.65	26.43	38.66
158.73	26.43	35.71

Table S5. Test size records of *Oridorsalis umbonatus* from ref. [18].

Revised composite depth (m)	Age (Ma)	Test size of *O. umbonatus*	Revised composite depth (m)	Age (Ma)	Test size of *O. umbonatus*
146.720	26.023	642.68	149.240	26.099	655.06
146.760	26.024	564.08	149.280	26.100	600.20
146.840	26.027	670.22	149.320	26.102	807.08
146.880	26.028	601.38	149.360	26.103	592.76
146.920	26.029	460.23	149.400	26.104	551.21
146.960	26.030	580.02	149.480	26.107	751.86
147.000	26.032	630.28	149.520	26.108	646.18
147.040	26.033	583.31	149.560	26.109	729.98
147.100	26.035	455.05	149.590	26.110	449.67
147.140	26.036	604.95	149.670	26.112	400.41
147.180	26.037	419.53	149.710	26.113	470.63
147.340	26.042	514.93	149.750	26.115	562.12
147.460	26.046	353.96	149.870	26.118	643.09
147.500	26.047	538.61	149.910	26.120	460.20
147.540	26.048	595.34	150.260	26.130	601.97
147.580	26.049	585.11	150.300	26.131	513.94
147.620	26.050	581.04	150.340	26.132	481.21
147.660	26.052	580.86	150.420	26.135	535.52
147.740	26.054	645.31	150.500	26.137	550.54
147.820	26.056	862.52	150.580	26.140	384.08
147.860	26.058	606.18	150.620	26.141	560.73
147.900	26.059	786.18	150.660	26.142	576.09
147.940	26.060	662.70	150.740	26.145	493.26
147.980	26.061	638.93	150.780	26.146	1079.18
148.020	26.062	900.71	150.820	26.147	400.10
148.060	26.064	925.38	150.900	26.149	575.22
148.100	26.065	660.68	150.940	26.151	628.07
148.140	26.066	584.85	150.980	26.152	771.03
148.180	26.067	547.59	151.020	26.153	724.37
148.220	26.068	565.70	151.060	26.154	639.59
148.260	26.070	413.15	151.100	26.155	803.30
148.300	26.071	664.67	151.140	26.157	603.30
148.340	26.072	427.51	151.180	26.158	863.57
148.380	26.073	575.52	151.220	26.159	866.43
148.420	26.075	700.37	151.260	26.160	800.43
148.460	26.076	451.55	151.300	26.161	665.25
148.500	26.077	881.03	151.340	26.163	657.67
148.540	26.078	514.73	151.380	26.164	815.11
148.600	26.080	622.18	151.420	26.165	464.39
148.640	26.081	517.68	151.460	26.166	616.11
148.680	26.082	633.39	151.500	26.167	601.60
148.720	26.084	538.78	151.540	26.169	596.00
148.760	26.085	469.60	151.580	26.170	563.71
148.800	26.086	830.64	151.620	26.171	838.19
148.840	26.087	601.63	151.700	26.174	745.84
148.880	26.088	615.85	151.740	26.175	675.50
148.920	26.090	621.88	151.780	26.176	481.02
148.960	26.091	511.44	151.820	26.177	402.76
149.000	26.092	610.04	152.020	26.183	437.61
149.040	26.093	486.03	152.140	26.187	460.30
149.200	26.098	589.43	152.180	26.188	597.41

Revised composite depth (m)	Age (Ma)	Test size of *O. umbonatus*	Revised composite depth (m)	Age (Ma)	Test size of *O. umbonatus*
152.220	26.189	588.81	155.390	26.285	449.35
152.300	26.192	656.41	155.510	26.289	404.04
152.340	26.193	574.41	155.670	26.293	473.30
152.380	26.194	652.48	155.710	26.295	476.26
152.420	26.195	745.80	155.750	26.296	492.49
152.540	26.199	618.71	155.790	26.297	675.24
152.580	26.200	770.02	155.830	26.298	949.01
152.620	26.201	531.14	155.910	26.301	438.63
152.660	26.203	660.96	155.950	26.303	428.29
152.700	26.204	705.68	156.030	26.307	783.83
152.740	26.205	513.01	156.110	26.310	531.53
152.820	26.207	558.49	156.230	26.316	580.12
152.860	26.209	620.79	156.390	26.323	538.53
152.900	26.210	548.41	156.430	26.325	469.27
152.940	26.211	955.62	156.510	26.329	491.05
152.980	26.212	660.52	156.550	26.331	516.49
153.020	26.213	913.14	156.590	26.333	405.12
153.060	26.215	848.83	156.700	26.338	456.68
153.100	26.216	810.36	156.740	26.340	418.10
153.140	26.217	931.15	156.780	26.342	484.78
153.230	26.220	872.46	156.820	26.344	380.26
153.270	26.221	576.64	156.860	26.345	596.96
153.310	26.222	622.63	156.900	26.347	423.65
153.350	26.223	443.43	156.940	26.349	397.08
153.390	26.225	461.32	156.980	26.351	398.17
153.430	26.226	420.88	157.060	26.355	497.46
153.750	26.235	464.38	157.100	26.357	542.82
153.790	26.237	738.96	157.140	26.359	772.84
153.800	26.237	403.65	157.220	26.362	657.95
153.920	26.241	821.44	157.260	26.364	701.83
154.040	26.244	853.13	157.300	26.366	435.28
154.080	26.245	939.55	157.340	26.368	536.91
154.120	26.247	463.63	157.380	26.370	448.28
154.160	26.248	1005.64	157.420	26.372	602.24
154.200	26.249	420.25	157.460	26.374	830.15
154.240	26.250	495.11	157.500	26.375	617.81
154.280	26.251	457.32	157.540	26.377	663.50
154.320	26.253	402.43	157.580	26.379	540.77
154.400	26.255	466.32	157.610	26.381	432.81
154.440	26.256	459.21	157.620	26.381	476.61
154.480	26.257	369.08	157.700	26.385	468.18
154.520	26.259	791.74	157.740	26.387	637.82
154.560	26.260	407.82	157.780	26.389	468.83
154.600	26.261	469.63	157.810	26.390	611.00
154.680	26.263	648.15	157.850	26.392	527.30
154.720	26.265	516.11	157.890	26.394	571.15
154.760	26.266	648.42	158.050	26.401	451.75
154.840	26.268	414.20	158.090	26.403	457.24
154.960	26.272	441.72	158.170	26.407	403.25
155.000	26.273	446.98	158.210	26.409	595.90
155.080	26.276	516.98	158.290	26.412	744.00
155.190	26.279	550.85	158.330	26.414	507.71
155.270	26.281	560.59	158.370	26.416	550.91
155.310	26.282	831.21	158.410	26.418	442.38

Revised composite depth (m)	Age (Ma)	Test size of *O. umbonatus*	
158.450	26.420	501.69	
158.490	26.422	587.49	
158.530	26.423	437.53	
158.570	26.425	811.64	
158.610	26.426	484.99	
158.650	26.428	520.02	
158.690	26.429	473.59	
158.730	26.431	517.34	

Supplementary references

1. Hauptvogel, D. W., Pekar, S. F., & Pincay, V. Evidence for a heavily glaciated Antarctica during the late Oligocene "warming" (27.8-24.5 Ma): Stable isotope records from ODP Site 690. *Paleoceanography* **32,** 384-396 (2017).

2. Villa, G., & Persico, D. Late Oligocene climatic changes: Evidence from calcareous nannofossils at Kerguelen Plateau Site 748 (Southern Ocean). *Palaeogeogr. Palaeoclim. Palaeoecol*. **231,** 110-119 (2006).

3. Pekar, S. F., Deconto, R. M., & Harwood, D. M. Resolving a late Oligocene conundrum: Deep-sea warming and Antarctic glaciation. *Palaeogeogr. Palaeoclim. Palaeoecol*. **231,** 29-40 (2006).

4. Vahlenkamp, M. *et al*. Astronomically paced changes in deep-water circulation in the western North Atlantic during the middle Eocene. *Earth Planet. Sci. Lett.* **484,** 329-340 (2018).

5. Norris, R. D. *et al*. Site U1406. In Norris, R. D., Wilson, P. A., Blum, P., and the Expedition 342 Scientists. *Proc. IODP* **342,** College Station, Texas, doi: 10.2204/iodp.proc.342.107.2014 (2014).

6. Egger, L. M. *et al*. Magnetostratigraphically-calibrated dinoflagellate cyst bioevents for the uppermost Eocene to lowermost Miocene of the western North Atlantic (IODP Expedition 342, Paleogene Newfoundland sediments drifts). *Rev. Palaeobot. Palynol.* **234,** 159-185, doi: dx.doi.org/10.1016/j.revpalbo.2016.08.002 (2016).

7. Schulz, M., & Mudelsee, M. REDFIT: estimating red-noise spectra directly from unevenly spaced paleoclimatic time series. *Comput. Geosci.* **28,** 421-426 (2002).

8. Expedition 342 Scientists. Paleogene Newfoundland sediment drifts. *IODP Prel. Rept.* **342,** doi: 10.2204/iodp.pr.342.2012 (2012).

9. Laskar, J. *et al*. A long-term numerical solution for the insolation quantities of the Earth. *Astron. Astrophys.* **428,** 261-285, doi: https://doi/org/10.1051/0004-6361:20041335 (2004).

10. Livermore, R., Hillenbrand, C-D., Meredith, M., & Eagles, G. Drake Passage and Cenozoic climate: An open and shut case?. Geoche. Geophys. Geosyst. **8** (2007).

11. Borrelli, C., Cramer, B. S., & Katz, M. E. Bipolar Atlantic deepwater circulation in the middle-late Eocene: Effects of Southern Ocean gateway openings. *Paleoceanography* **29,** doi:10.1002/2012PA002444 (2014).

12. Scher, H. D., & Martin, E. E. Timing and climatic consequences of the opening of Drake Passage. *Science* **312,** 428-430 (2006).

13. Scher, H. D. *et al*. Onset of Antarctic Circumpolar Current 30 million years ago as Tasmanian Gateway aligned with westerlies. *Nature* **523,** 580-583 (2015).

14. Lee, H., & Jo, K.-n. Oligocene paleoceanographic changes based on an interbasinal comparison of *Cibicidoides* spp. δ^{18}O records and a new compilation of data. *Palaeogeogr. Palaeoclim. Palaeoecol*. **514,** 800-812 (2019).

15. Abelson, M., Agnon, A., & Almogi-Labin, A. Indications for control of the Iceland plume on the Eocene-Oligocene “greenhouse-icehouse” climate transition. Earth Planet. Sci. Lett. **265,** 33-48 (2008).

16. Billups, K., Channell, J. E. T., & Zachos, J. C. Late Oligocene to early Miocene geochronology and paleoceanography from the subantarctic South Atlantic. *Paleoceanography* **17,** doi:10.1029/2000PA000568 (2002).

17. Shackleton, N. J., Hall, M. A., & Noersma, A. Oxygen and carbon isotope data from Leg 74 foraminifers. In: Moore, T. C., Jr., Rabinowitz, P. D., *et al*., *Init. Repts. DSDP* **74,** Washington (1984).

18. Lee, H., Jo, K.-n., & Lim, J. The strengthening of North Atlantic Deep Water during the late Oligocene based on the benthic foraminiferal species *Oridorsalis umbonatus*. *J. Geol. Soc. Korea.* **54,** 489-499 (2018) (in Korean with English abstract).

The English in this document has been checked by at least two professional editors, both native speakers of English. For a certificate, please see:

http://www.textcheck.com/certificate/eU3jCt
